# Synthesis of triphenylpyridines *via* an oxidative cyclization reaction using Sr-doped LaCoO_3_ perovskite as a recyclable heterogeneous catalyst†

**DOI:** 10.1039/c9ra04096j

**Published:** 2019-08-01

**Authors:** Thu N. M. Le, Son H. Doan, Phuc H. Pham, Khang H. Trinh, Tien V. Huynh, Tien T. T. Tran, Minh-Vien Le, Tung T. Nguyen, Nam T. S. Phan

**Affiliations:** Faculty of Chemical Engineering, HCMC University of Technology, VNU-HCM 268 Ly Thuong Kiet, District 10 Ho Chi Minh City Vietnam tungtn@hcmut.edu.vn ptsnam@hcmut.edu.vn +84 8 38637504 +84 8 38647256 extn 5681

## Abstract

An La_0.6_Sr_0.4_CoO_3_ strontium-doped lanthanum cobaltite perovskite was prepared *via* a gelation and calcination approach and used as a heterogeneous catalyst for the synthesis of triphenylpyridines *via* the cyclization reaction between ketoximes and phenylacetic acids. The transformation proceeded *via* the oxidative functionalization of the sp^3^ C–H bond in phenylacetic acid. The La_0.6_Sr_0.4_CoO_3_ catalyst demonstrated a superior performance to that of the pristine LaCoCO_3_ as well as a series of homogeneous and heterogeneous catalysts. Furthermore, the La_0.6_Sr_0.4_CoO_3_ catalyst was facilely recovered and reused without considerable decline in its catalytic efficiency. To the best of our knowledge, the combination of ketoximes with easily available phenylacetic acids is novel.

## Abstract 2

An La_0.6_Sr_0.4_CoO_3_ strontium-doped lanthanum cobaltite perovskite was prepared *via* a gelation and calcination approach and used as a heterogeneous catalyst for the synthesis of triphenylpyridines *via* the cyclization reaction between ketoximes and phenylacetic acids.

## Graphical abstract

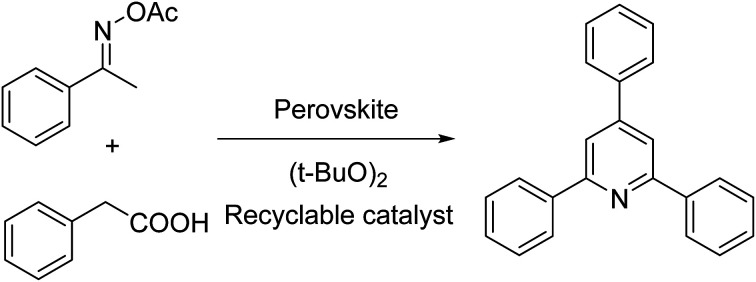

## 1. Introduction

**Scheme 1:**
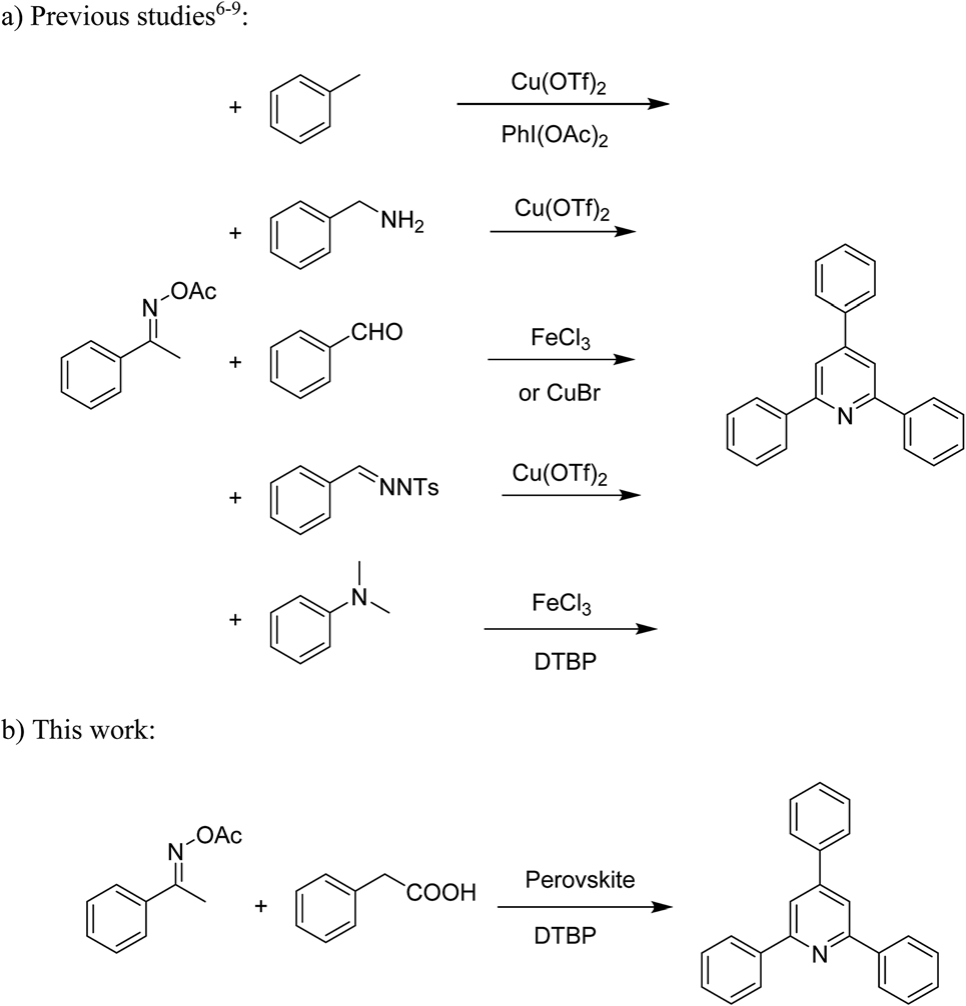
The difference between previous works (a) and this work (b).

Functionalized pyridines have emerged as a precious class of privileged *N*-heterocyclic compounds, existing in a wide range of biologically active natural products, medicinal chemicals, agricultural chemicals, and functional organic materials.^1–4^ Among the numerous pyridines, 2,4,6-triphenylpyridines offer important applications, and the development of effective synthetic approaches for these structures has been considered as a hot topic. The most popular method relies on the three-component cyclocondensation transformation of acetophenones, benzaldehydes, and ammonium acetate under diverse conditions.^5^ Recently, new synthetic protocols have been developed, in which the utilization of ketoximes as starting materials has been explored for the synthesis of the abovementioned heterocyclic compounds. Ren *et al.* developed an efficient strategy to achieve 2,4,6-triphenylpyridines *via* the CuBr-catalyzed coupling of oxime acetates with aldehydes.^6^ Fu reported the Cu(OTf)_2_-catalyzed oxidative sp^3^ C–H coupling of oxime acetates with toluene, benzylamines, and *p*-toluenesulfonylhydrazones.^7^ Iron(iii) chloride was used for the cyclization of ketoxime carboxylates with *N*,*N*-dialkylanilines or aldehyde to afford densely substituted pyridines^8,9^ (Scheme 1a). The coupling of aryl amines or benzyl halides and aromatic ketones for the synthesis of triarylpyridines is also known.^10–12^ Certainly, ketoximes have recently gained significant interest as valuable building blocks for numerous important organic structures.^13–17^

Perovskites have attracted extensive interest from researchers worldwide owing to their significant physicochemical properties, such as redox behavior, superconductivity, ferroelectricity, magnetoresistance, and ionic conductivity.^18–20^ Perovskites are mixed oxides, generally existing in the form of ABO_3_, where A represents an alkali, alkaline or lanthanide metal, and B stands for a transition metal.^21–23^ The metal A is responsible for the stability of the perovskite structure, and the substitution of A by other metals affects the valence state of metal B, leading to different catalytic activities.^24^ Certainly, perovskites have been extensively utilized as heterogeneous catalysts for numerous reduction–oxidation processes.^25–31^ LaCoO_3_-based perovskites are one of the most active catalysts for CO oxidation,^24,32^ NO oxidation,^33^ syngas conversion,^34,35^ and hydrogen production.^36^ Nonetheless, the application of perovskite catalysts in the organic synthesis field is extremely rare in the literature. Additionally, the utilization of easily available phenylacetic acids as green starting materials for organic synthesis *via* the oxidative functionalization of sp^3^ C–H bonds has gained considerable attention.^37–39^ In this work, we report the utilization of a strontium-doped lanthanum cobaltite perovskite as a recyclable heterogeneous catalyst for the synthesis of triphenylpyridines *via* oxidative cyclization reaction between ketoximes and phenylacetic acids (Scheme 1b). Single oxides or salts of lanthanum, strontium, and cobalt demonstrated a low catalytic performance for the transformation, indicating the importance of the synergistic effect in the perovskite catalyst. To the best of our knowledge, the oxidative cyclization between ketoximes and phenylacetic acids has not been previously mentioned in any literature.

## 2. Experimental

The strontium-doped lanthanum cobaltite perovskite (La_0.6_Sr_0.4_CoO_3_) powder was prepared from La(NO_3_)_3_, Sr(NO_3_)_2_, and Co(NO_3_)_2_*via* a gelation and calcination approach, as previously reported in the literature.^33^ The perovskite was accordingly characterized using several conventional analysis techniques (Fig. S1–S4†). In a representative experiment, a mixture of acetophenone oxime acetate (0.106 g, 0.6 mmol), phenylacetic acid (0.027 g, 0.2 mmol), and diphenyl ether (16 μL, 0.1 mmol) as an internal standard was added to a 12 mL screw-cap pressurized vial containing toluene (2 mL). The reaction mixture was magnetically stirred and heated for 5 min. The La_0.6_Sr_0.4_CoO_3_ perovskite catalyst (2.3 mg, 5 mol%) was then added to the vial. The reaction mixture was magnetically stirred for 2 min to completely disperse the catalyst in the liquid phase, followed by the addition of di-*tert*-butylperoxide (DTBP, 0.14 mL, 0.6 mmol). The resulting mixture was stirred at 140 °C for 2 h. Subsequently, the reactor tube was back-filled with argon and then stirred at 140 °C for 6 h. The reaction mixture was diluted with ethyl acetate (5 mL) and washed with saturated NaHCO_3_ solution (5 mL). The organic layer was dried using anhydrous Na_2_SO_4_. Reaction yields were recorded from the GC analysis results based on the diphenyl ether internal standard. To isolate the triphenylpyridine product, the ethyl acetate phase was concentrated under reduced pressure, and purified by column chromatography on silica gel with a hexane/ethyl acetate solvent mixture. The triphenylpyridine was consequently confirmed by GC-MS, ^1^H NMR, and ^13^C NMR. The strontium-doped lanthanum cobaltite perovskite catalyst was collected from the reaction mixture by centrifugation, washed carefully with ethyl acetate, acetone, and diethyl ether, dried under reduced pressure at room temperature overnight on a Schlenk line, and reutilized in the catalyst recycling studies.

## 3. Results and discussion

The strontium-doped lanthanum cobaltite perovskite was used as a heterogeneous catalyst for the synthesis of 2,4,6-triphenylpyridine *via* the oxidative cyclization reaction between acetophenone oxime acetate and phenylacetic acid (Scheme 1b). The reaction proceeded in the presence of an oxidant, which is required for the oxidative functionalization of the sp^3^ C–H bond in phenylacetic acid. Initially, the reaction conditions were optimized to improve the yield of the triphenylpyridine product (Table 1). For the reactions conducted in the liquid phase, the nature of the solvent may be critical, especially when a solid catalyst is employed. Thus, the impact of solvent on the yield of 2,4,6-triphenylpyridine was studied. Running the reaction in chlorobenzene gave 54% yield of the substituted pyridine (entry 1). Heptane was inferior to toluene (entries 2 and 3). It was noted that polar aprotic solvents such as DMF, DMAc, DMSO, and NMP were almost ineffective for the reaction, with less than 5% yield recorded (see the ESI† for details).

**Table 1:** Studies of reaction conditions^a^

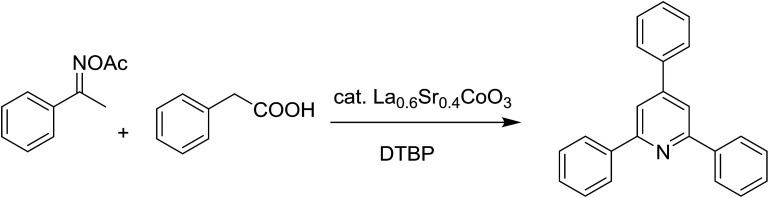				
Entry	Solvent	Catalyst amount (mol%)	Oxidant	Yield^b^ (%)
1	Chlorobenzene	5	DTBP	54
2	Heptane	5	DTBP	22
3	Toluene	5	DTBP	83
4^c^	Toluene	5	DTBP	17
5	Toluene	0	DTBP	4
6	Toluene	1	DTBP	21
7	Toluene	3	DTBP	51
8	Toluene	7	DTBP	87
9	Toluene	5	K_2_S_2_O_8_	8
10	Toluene	5	AgNO_3_	11
11	Toluene	5	Oxygen	2
12^d^	Toluene	5	TBHP	55

a Reaction conditions: acetophenone oxime acetate (0.6 mmol), phenylacetic acid (0.2 mmol), solvent (2 mL), oxidant (0.6 mmol), at 140 °C under air for 2 h and under argon for 6 h.

b GC yield.

c Reaction at 120 °C.

d TBHP in water. DTBP = di-*tert*-butylperoxide, TBHP = *tert*-butyl hydroperoxide.

With these results, the influence of temperature on the reaction was explored. The reaction was conducted at different temperatures, ranging from room temperature to 140 °C. No product was detected when the temperature was lower than 120 °C, and 17% yield was detected for the reaction performed at 120 °C (entry 4). Additionally, the effect of the catalyst amount on the yield of 2,4,6-triphenylpyridine was investigated. Only 4% yield was detected in the absence of the strontium-doped lanthanum cobaltite perovskite catalyst, indicating that the perovskite was critical for the oxidative cyclization reaction (entry 5). Using 1 mol% catalyst, the reaction afforded 21% yield (entry 6). As expected, increasing the perovskite quantity enhanced the yield of the triphenylpyridine product. Extending the catalyst amount to 3 mol% led to 51% yield, while 87% yield was achieved in the presence of 7 mol% catalyst (entries 7 and 8, respectively).

An oxidant should be present for the oxidative functionalization of the sp^3^ C–H bond in phenylacetic acid, which facilitates the cyclization.^37^ Accordingly, the impact of oxidant on the yield of 2,4,6-triphenylpyridine was addressed. The experiment was conducted under standard conditions, using a variety of inorganic and organic oxidants. The reaction using K_2_S_2_O_8_ as the oxidant gave a sluggish crude mixture, with only 8% yield (entry 9). Similarly, AgNO_3_ and oxygen were not suitable for this reaction (entries 10 and 11, respectively). *tert*-Butyl hydroperoxide in water was somewhat effective, affording the triphenylpyridine product in 55% yield (entry 12).

**Fig. 1:**
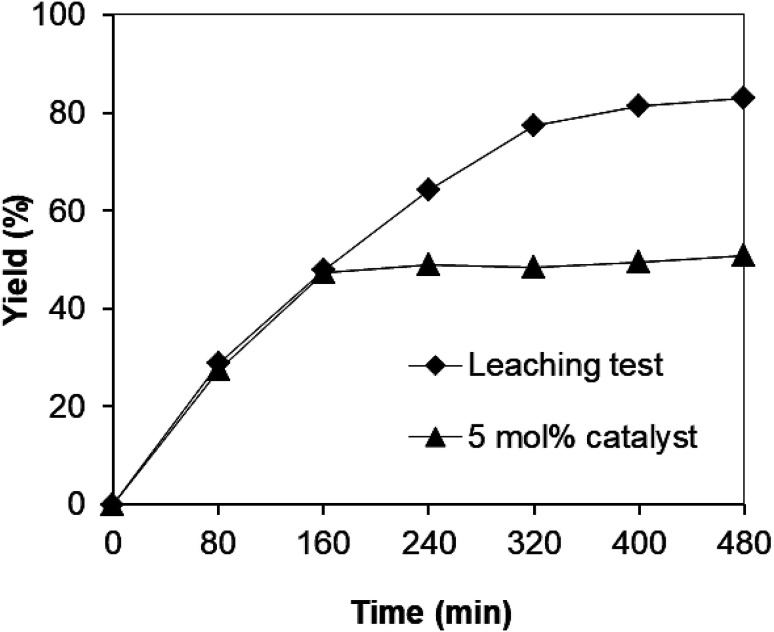
Leaching experiment indicating that no additional 2,4,6-triphenylpyridine was formed after the removal of the catalyst.

Since the synthesis of 2,4,6-triphenylpyridine *via* oxidative cyclization between acetophenone oxime acetate and phenylacetic acid was conducted in toluene, a critical issue is that the leached species from the strontium-doped lanthanum cobaltite perovskite may be accountable a considerable part of the catalytic efficiency. Thus, to clarify if leaching was serious in this reaction, a control experiment was performed. The reaction was carried out in toluene under argon at 140 °C for 8 h using 3 equivalents of ketoxime and 3 equivalents of DTBP in the presence of 5 mol% catalyst. After the first 160 min reaction time with 47% yield of 2,4,6-triphenylpyridine recorded, the perovskite catalyst was removed by centrifugation. The reaction solution was transferred to a new 12 mL screw-cap pressurized vial. The reaction vial was back-filled with argon and the resulting mixture was subsequently stirred at 140 °C for an additional 320 min. Aliquots were taken at different time intervals, and subsequently analyzed by GC. The GC data provided quantitative information regarding the residual, catalytically active sites in the toluene phase. It was observed that almost no additional 2,4,6-triphenylpyridine was generated in the absence of the strontium-doped lanthanum cobaltite perovskite (Fig. 1). These results verified that the perovskite-catalyzed oxidative cyclization between acetophenone oxime acetate and phenylacetic acid proceeded under truly heterogeneous conditions.

To emphasize the advantages of using the strontium-doped lanthanum cobaltite perovskite catalyst, its activity in the oxidative cyclization reaction was compared to that of other catalysts (Table 2). The reaction was conducted in toluene at 140 °C for 8 h, using 3 equivalents of ketoxime and 3 equivalents of DTBP, in the presence of 5 mol% catalyst. First, traditional homogeneous catalysts were utilized for the reaction (entries 1–10). The FeCl_3_-catalyzed transformation afforded 26% yield, while 6% yield was observed for the reaction using the FeCl_2_ catalyst. Fe(NO_3_)_3_ and FeSO_4_ displayed a low catalytic performance for the reaction, with 3% and 13% yields being detected, respectively. In the case of copper salts, the use of CuSO_4_ resulted in 57% yield, while 8% yield was noted for the reaction using a Cu(NO_3_)_2_ catalyst. NiSO_4_ demonstrated a low catalytic performance, although 38% yield was recorded. The three precursors used to synthesize the strontium-doped lanthanum cobaltite perovskite catalyst, including La(NO_3_)_3_, Sr(NO_3_)_2_, and Co(NO_3_)_2_, also exhibited low catalytic activity, forming the expected product in 2%, 39%, and 4% yields, respectively. In a second series of experiments, several heterogeneous catalysts were utilized for the reaction (entries 11–18). Cu(BDC), a copper–organic framework, was active for the reaction, producing the product in 70% yield. The reaction using a ZIF-8A catalyst, a zinc–organic framework, progressed to 60% yield. UiO-66·TFA, a zirconium–organic framework, was less reactive toward the oxidative cyclization reaction, generating the desired product in 40% yield. The single oxides La_2_O_3_, CoO, and SrO exhibited low activity, though 38% yield was detected for the case of SrO (entries 14–16). The pristine perovskite, LaCoCo_3_, was also not very active towards the transformation, although 40% yield was detected (entry 17). Interestingly, using the strontium-doped perovskite La_0.6_Sr_0.4_CoO_3_ as a catalyst for the oxidative cyclization reaction led to 83% yield (entry 18).

**Table 2:** The oxidative cyclization between acetophenone oxime acetate and phenylacetic acid utilizing different catalysts^a^

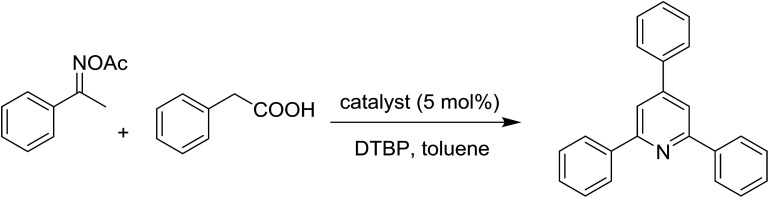			
Entry	Homogeneous catalyst	Heterogeneous catalyst	Yield^b^ (%)
1	FeCl_3_		26
2	FeCl_2_		6
3	Fe(NO_3_)_3_		3
4	FeSO_4_		13
5	CuSO_4_		57
6	Cu(NO_3_)_2_		8
7	NiSO_4_		38
8	Co(NO_3_)_2_		4
9	La(NO_3_)_2_		2
10	Sr(NO_3_)_2_		39
11		Cu(BDC)	70
12		ZIF-8A	60
13		UiO-66·TFA	43
14		La_2_O_3_	19
15		CoO	11
16		SrO	38
17		LaCoCo_3_	40
18		La_0.6_Sr_0.4_CoO_3_	83

a Reaction conditions: acetophenone oxime acetate (0.6 mmol), phenylacetic acid (0.2 mmol), DTBP (0.6 mmol), toluene (2 mL), catalyst (5 mol%), 140 °C, under air for 2 h and under argon for 6 h.

b GC yield.

**Scheme 2:**
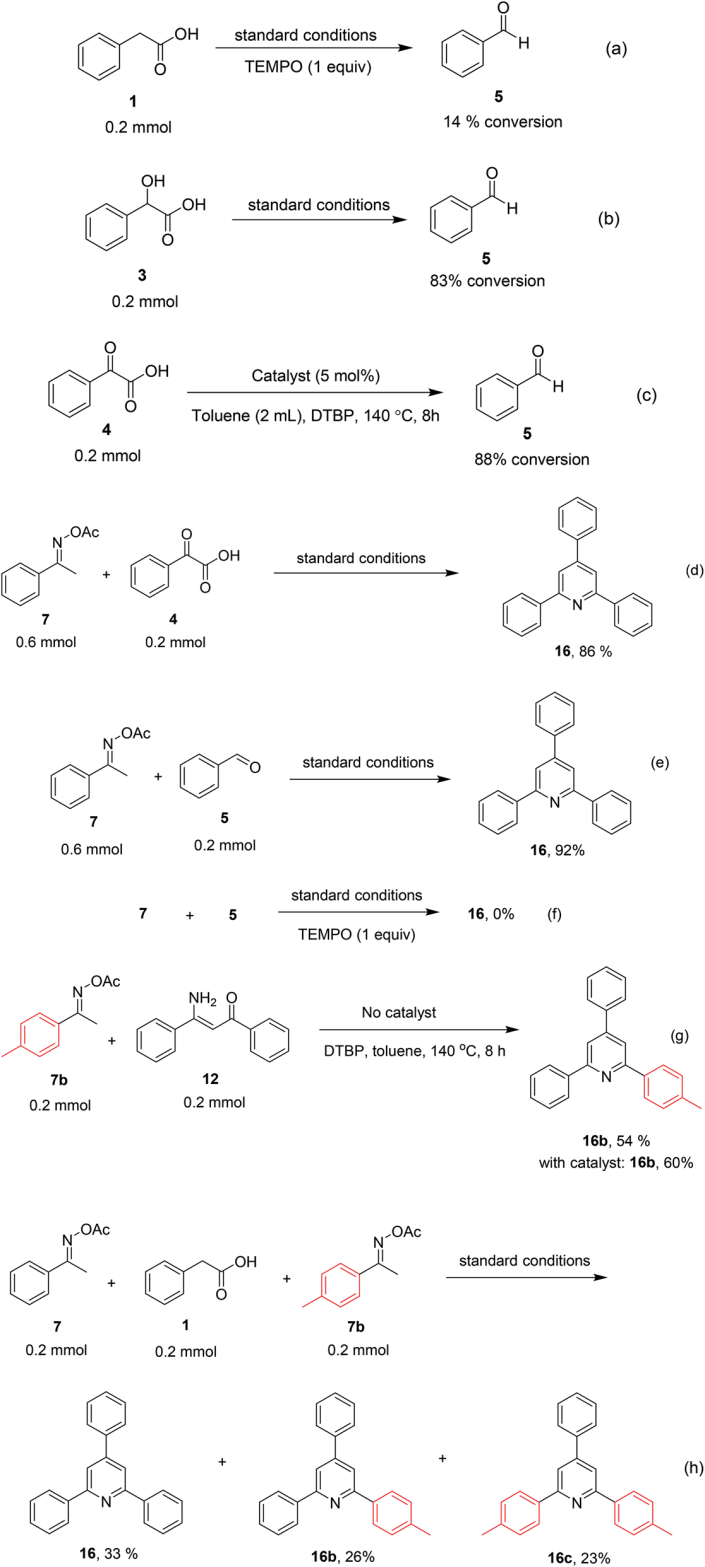
Control experiments.

To comprehend the mechanism of the oxidative cyclization reaction, several control experiments were performed, as highlighted in Scheme 2. The presence of (2,2,6,6-tetramethylpiperidin-1-yl)oxy (TEMPO) as a radical scavenger had a significant effect on the formation of benzaldehyde (5) in the first step, which implied the involvement of a radical process in the oxidative decarboxylation of phenylacetic acid (1) (Scheme 2a). In the next two experiments, high conversions to benzaldehyde were achieved, which indicated that 2-hydroxy-2-phenylacetic acid (3) and 2-oxo-2-phenylacetic acid (4) were the likely intermediates of this process (Scheme 2b and c, respectively). When the oxidative cyclization reaction between ketoxime (7) and 2-oxo-2-phenylacetic acid (4) was subsequently carried out under standard conditions, 2,4,6-triphenylpyridine (16) was obtained in 86% yield, suggesting that 2-oxo-2-phenylacetic acid may be an important intermediate (Scheme 2d). The cyclization of the ketoxime (7) and benzaldehyde (5) generated 2,4,6-triphenylpyridine (16) in high yield (92%), which demonstrates that benzaldehyde (5) may be the key intermediate in this reaction (Scheme 2e). The reaction between the ketoxime (7) and benzaldehyde (5) did not proceed in the presence of the antioxidant TEMPO, indicating that a radical process may be involved and that the interaction of TEMPO with the radical species generated in the catalytic cycle may have cease the transformation (Scheme 2f). (*Z*)-3-Amino-1,3-diphenylprop-2-en-1-one (12) was synthesized and allowed to undergo the cyclization with the ketoxime (7) under two different conditions. The yield of 2,4,6-triphenylpyridine in the cyclization without the La_0.6_Sr_0.4_CoO_3_ catalyst was similar to that of the cyclization using the La_0.6_Sr_0.4_CoO_3_ catalyst (Scheme 2g). The reaction between (1), (7), and (7b) produced 3 products (16, 16b, and 16c) in similar yields under the standard conditions (Scheme 2h). These results suggest that (*Z*)-3-amino-1,3-diphenylprop-2-en-1-one (12) may be the intermediate in this reaction, and that the transformation of (*Z*)-3-amino-1,3-diphenylprop-2-en-1-one (12) to 2,4,6-triphenylpyridine (16) could proceed in the absence of the La_0.6_Sr_0.4_CoO_3_ catalyst.

**Scheme 3:**
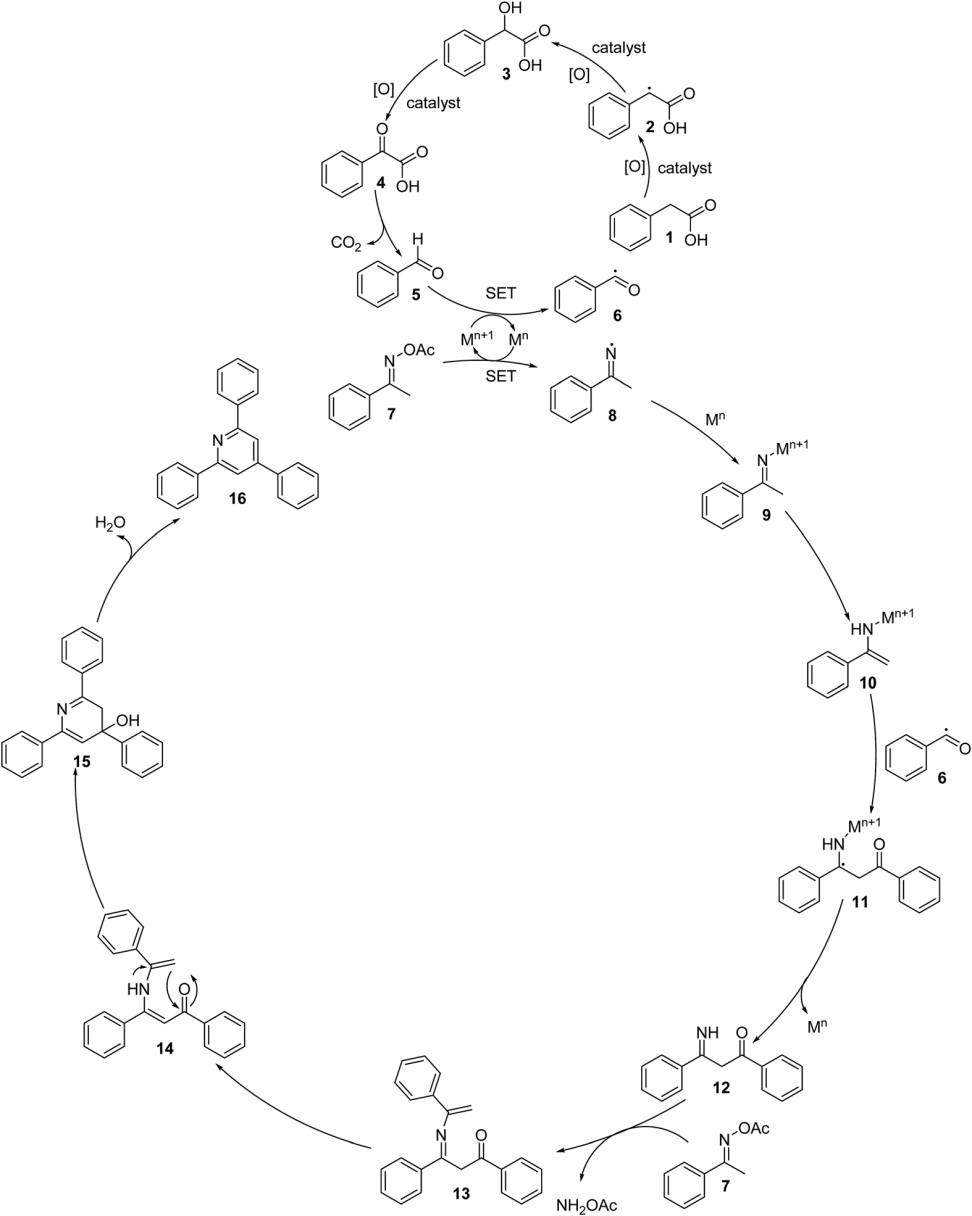
Proposed reaction mechanism.

Based on the aforementioned results and previous reports,^8,37,40–43^ a plausible mechanism is proposed in Scheme 3. The first step is the aerobic oxidation of phenylacetic acid (1) to benzaldehyde (5) *via* the formation of 2-hydroxy-2- phenylacetic acid (3) and 2-oxo-2-phenylacetic acid (4), followed by the single electron transfer (SET) process between benzaldehyde and M^*n*+1^ to generate benzoyl radical (6). It should be noted that a benzylic radical (2) is formed in this process. The next step is the reductive cleavage of the N–O bond of the ketoxime (7) by M^*n*^*via* a single electron transfer (SET) to achieve the M^*n*+1^ species and imine radical (8), which undergo rapid coordination to M^*n*^ to give the imino-M^*n*+1^ intermediate (9). Tautomerization of the imino-M^*n*+1^ intermediate (9) forms the enamino-M^n+1^ intermediate (10), which reacts with the benzoyl radical (6) to generate a new radical (11). This radical undergoes a single electron transfer (SET) process, resulting in the formation of intermediate (12) upon regeneration of the M^*n*^ species. Subsequently, the condensation of the intermediate (12) with a second molecule of the ketoxime (7) produces the intermediate (13) and releases NH_2_OAc. Tautomerization of intermediate (13) affords intermediate (14), followed by the intramolecular cyclization of intermediate (14), generating intermediate (15). Finally, the dehydration of intermediate (15) produces 2,4,6-triphenylpyridine (16). In this catalytic cycle, M is the cobalt species in the La_0.6_Sr_0.4_CoO_3_ catalyst. It should be noted that using CoO as a catalyst for the oxidative cyclization reaction resulted in a very low yield (entry 15, Table 2). The pristine perovskite, LaCoCo_3_, was also not very active towards the transformation (entry 17, Table 2), while utilizing the strontium-doped perovskite, La_0.6_Sr_0.4_CoO_3_ as the catalyst afforded 80% yield (entry 18, Table 2). It was previously reported that substituting the La(iii) site by Sr(ii) site in the perovskite will improve the mobility of lattice oxygen, thus significantly enhancing the redox activity of the catalyst.^24,44^ Certainly, the synergistic effect in the La_0.6_Sr_0.4_CoO_3_ catalyst is critical for the oxidative cyclization reaction.

**Fig. 2:**
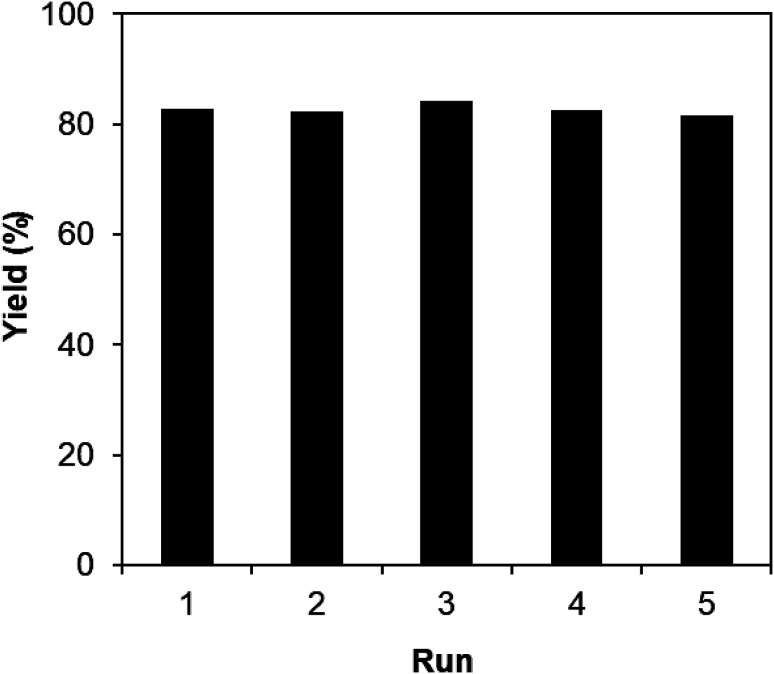
Reutilization of the La_0.6_Sr_0.4_CoO_3_ catalyst.

**Fig. 3:**
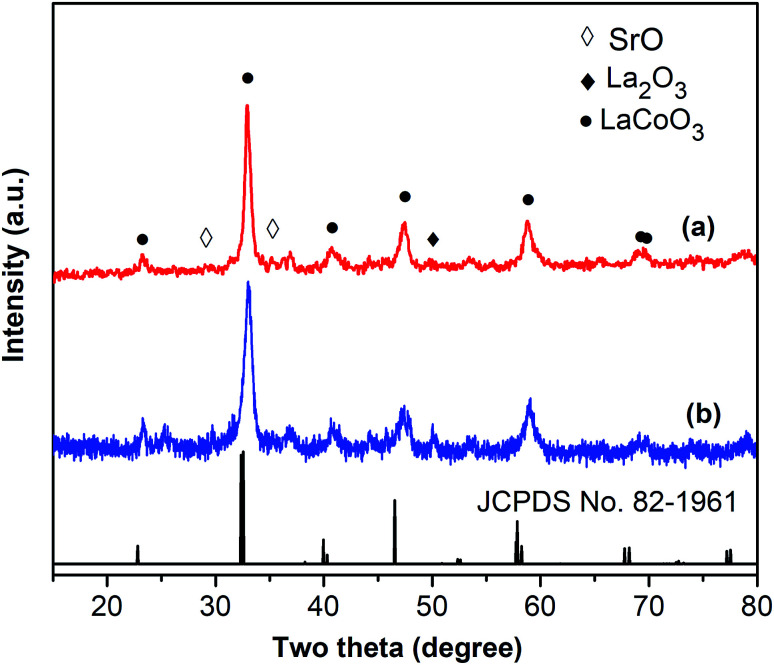
XRD results for the fresh (a) and reutilized (b) La_0.6_Sr_0.4_CoO_3_ perovskite catalysts.

**Fig. 4:**
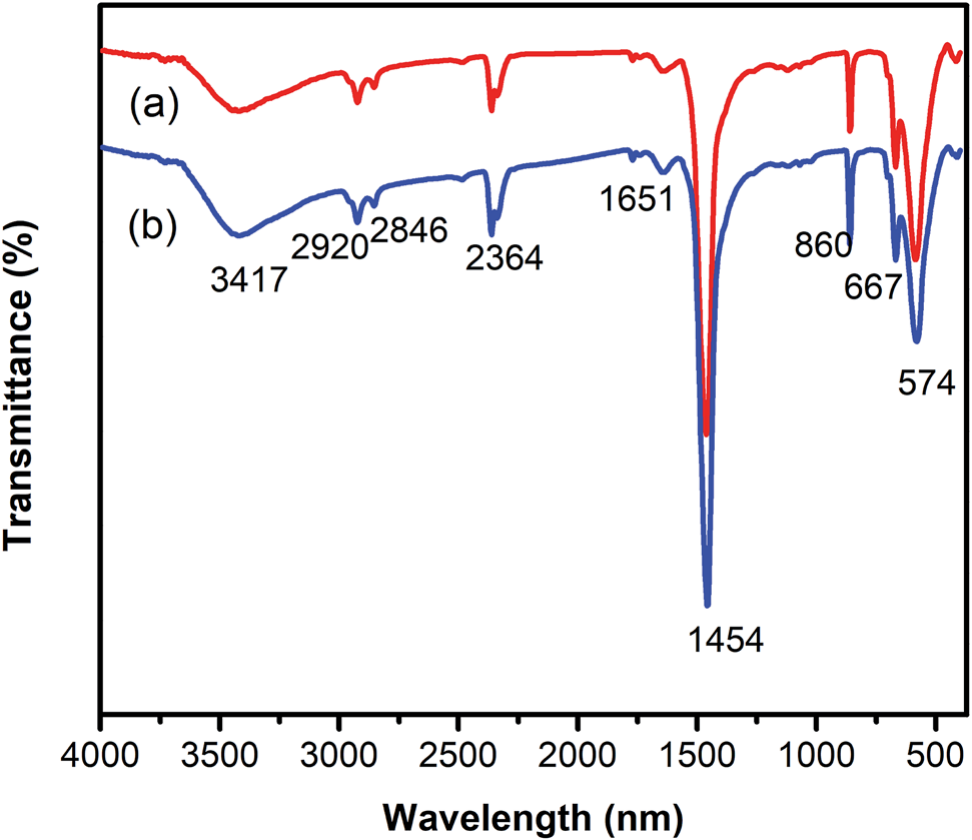
FT-IR observations for the fresh (a) and reutilized (b) La_0.6_Sr_0.4_CoO_3_ perovskite catalysts.

One critical aspect that must be addressed for the oxidative cyclization reaction using the strontium-doped lanthanum cobaltite perovskite catalyst is the possibility of catalyst reutilization. In the best case, it should be possible to recover and reutilize the solid catalyst many times before it ultimately becomes totally deactivated. Accordingly, the reutilization of the La_0.6_Sr_0.4_CoO_3_ catalyst was investigated for the synthesis of 2,4,6-triphenylpyridine *via* the oxidative cyclization reaction between acetophenone oxime acetate and phenylacetic acid under the standard conditions. The reaction was conducted in toluene at 140 °C for 8 h using 3 equivalents of ketoxime and 3 equivalents of DTBP, in the presence of 5 mol% catalyst. At the end of each catalytic run, the La_0.6_Sr_0.4_CoO_3_ catalyst was collected from the reaction mixture by centrifugation, washed carefully with ethyl acetate, acetone, and diethyl ether. The recovered catalyst was dried under reduced pressure at room temperature overnight on a Schlenk line, and reutilized for the next experiment. The experimental results indicated that the La_0.6_Sr_0.4_CoO_3_ catalyst could be reutilized several times in the oxidative cyclization reaction. At the fifth catalytic run, 82% yield of 2,4,6-triphenylpyridine was recorded (Fig. 2). Moreover, the recovered La_0.6_Sr_0.4_CoO_3_ catalyst was characterized *via* XRD and FT-IR analyses, and the results were compared with that of the new strontium-doped perovskite. The XRD (Fig. 3) and FT-IR (Fig. 4) observations revealed that the La_0.6_Sr_0.4_CoO_3_ catalyst was stable in the synthesis of 2,4,6-triphenylpyridine *via* the oxidative cyclization reaction under the standard conditions.

With these achievements, we consequently extended the scope of this work to the synthesis of several 2,4,6-triphenylpyridines *via* oxidative cyclization reactions between ketoximes and phenylacetic acids utilizing the La_0.6_Sr_0.4_CoO_3_ perovskite catalyst (Table 3). The reaction was conducted in toluene at 140 °C for 8 h using 3 equivalents of ketoxime and 3 equivalents of DTBP, in the presence of 5 mol% catalyst. At the end of the experiment, the triphenylpyridine product was purified by column chromatography. First, different acetophenone oxime acetates were employed for the oxidative cyclization with phenylacetic acid. Under these conditions, 2,4,6-triphenylpyridine was obtained in 79% yield (entry 1). Acetophenone oxime acetates possessing an electron-donating substituent on the benzene ring were also reactive towards the reaction. Under the standard conditions, 4-phenyl-2,6-di-*p*-tolylpyridine (entry 2), 2,6-bis(3-methoxyphenyl)-4-phenylpyridine (entry 3), and 2,6-bis(2-methoxyphenyl)-4-phenylpyridine (entry 4) were generated in 62%, 74%, and 67% yields, respectively. Similarly, acetophenone oxime acetates containing an electron-withdrawing substituent on the benzene ring were utilized, producing 2,6-bis(4-chlorophenyl)-4-phenylpyridine (entry 5), 2,6-bis(3-chlorophenyl)-4-phenylpyridine (entry 6), and 2,6-bis(4-bromophenyl)-4-phenylpyridine (entry 7) in 55%, 68%, and 73% yields, respectively. (*E*)-3,4-Dihydronaphthalen-1(2*H*)-one *O*-acetyl oxime was reactive, and the reaction afforded the corresponding product in 69% yield (entry 8). Using (*E*)-1-(thiophen-2-yl)ethan-1-one *O*-acetyl oxime led to 72% yield of the desired product (entry 9). Moving to phenylacetic acids containing a substituent, the oxidative cyclization reactions with acetophenone oxime acetate afforded the corresponding triphenylpyridine products in reasonable yields (entries 10–12). Heterocyclic acetic acids were also competent substrates, affording the products in moderate yields (entries 13–15).

**Table 3:** Synthesis of 2,4,6-triphenylpyridines *via* oxidative cyclization reaction between ketoximes and phenylacetic acids utilizing the La_0.6_Sr_0.4_CoO_3_ perovskite catalyst^a^

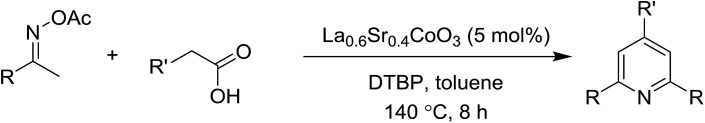				
Entry	Reactant 1	Reactant 2	Product	Yield^b^ (%)
1	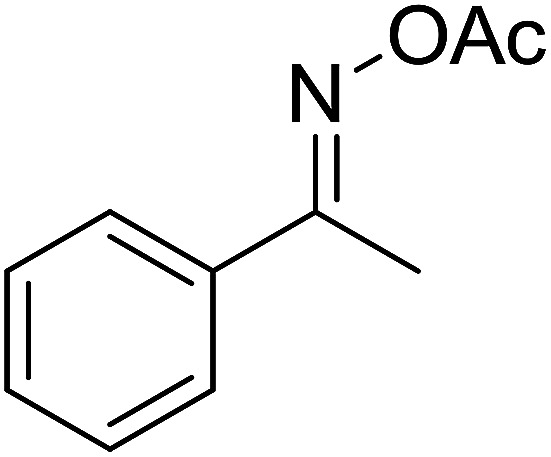	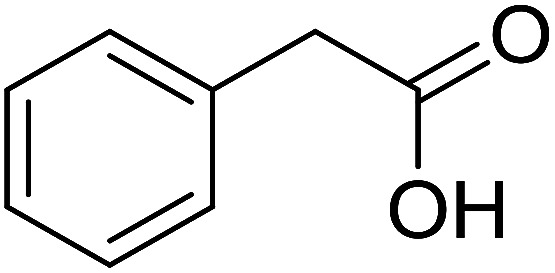	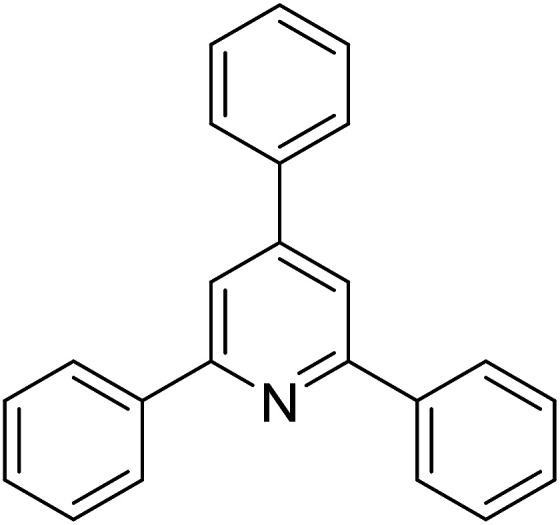	79
2	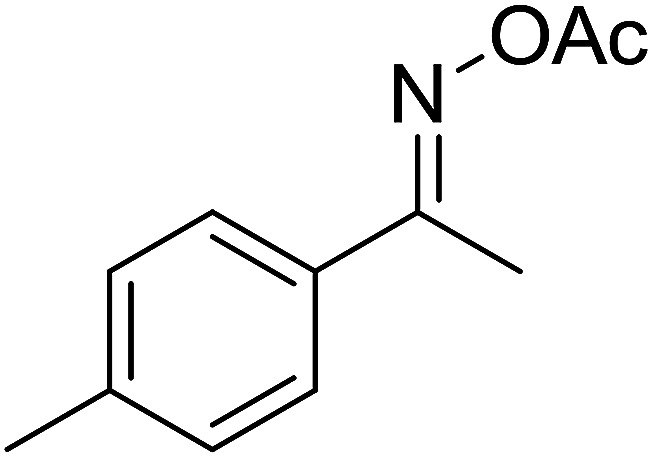	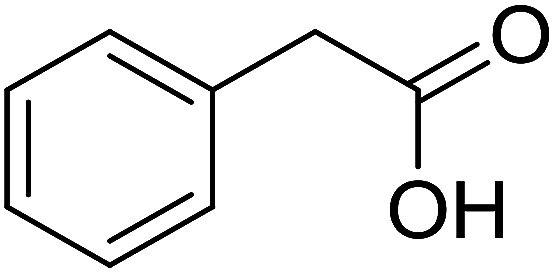	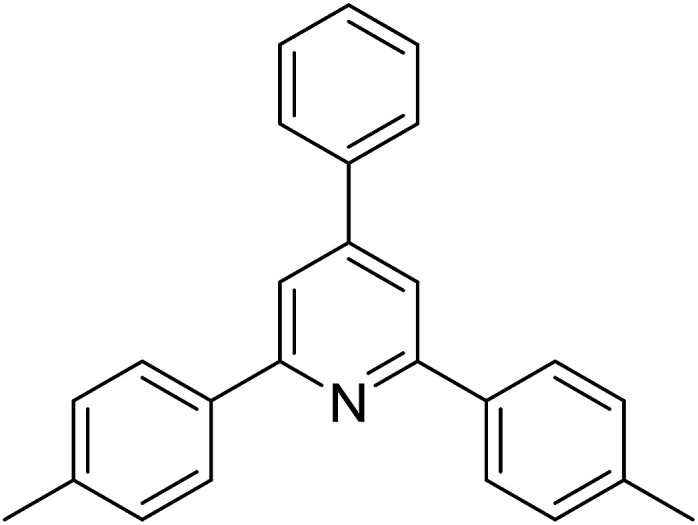	62
3	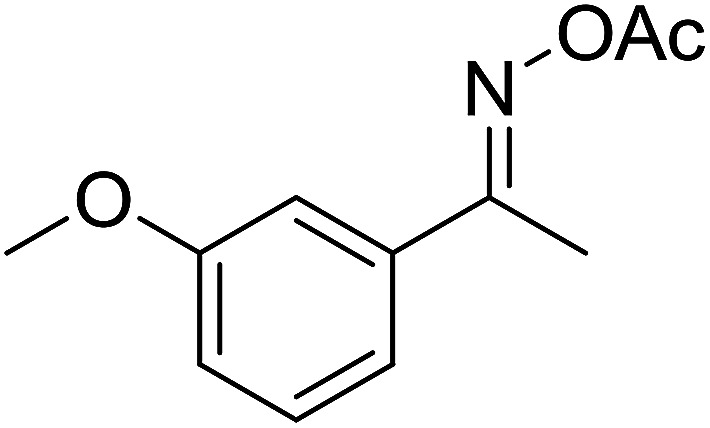	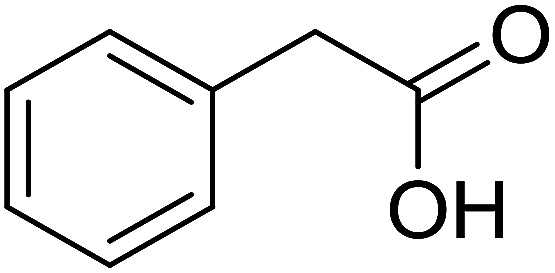	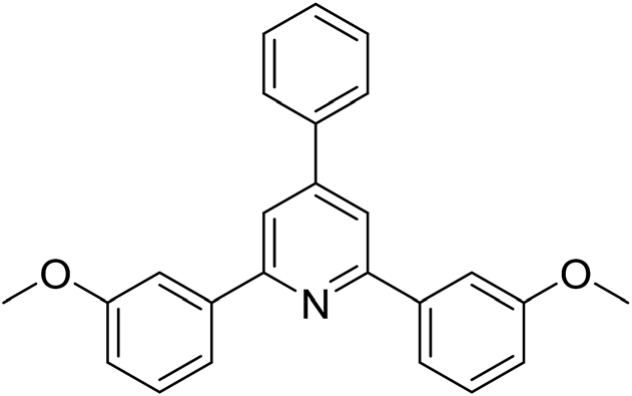	74
4	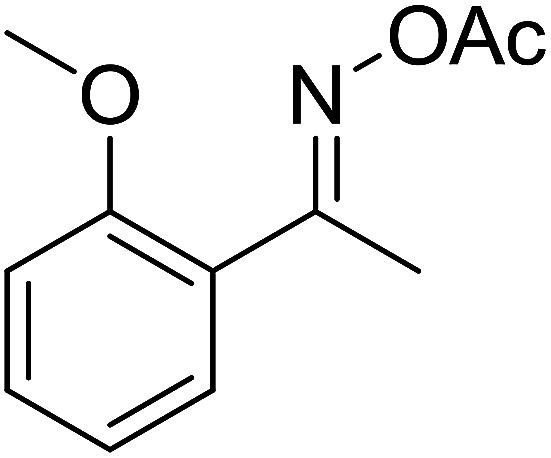	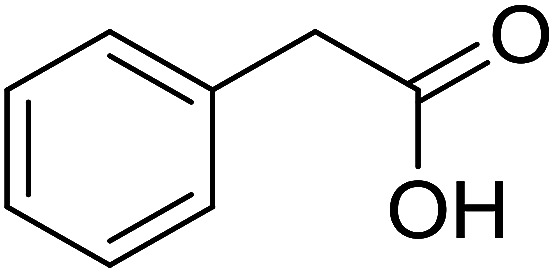	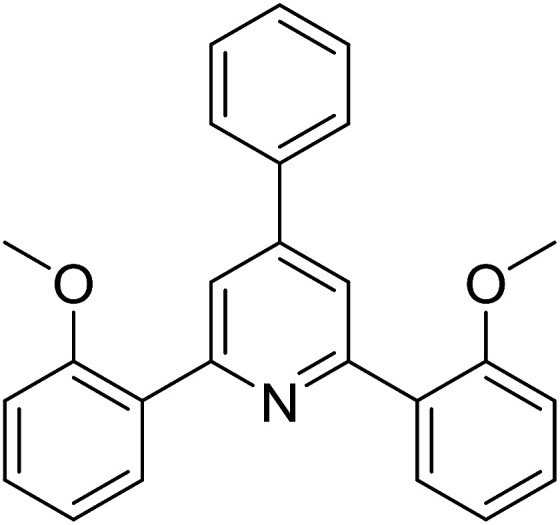	67
5	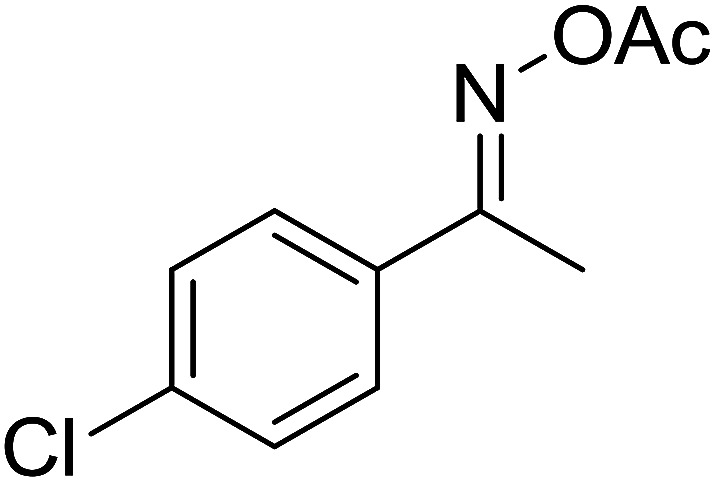	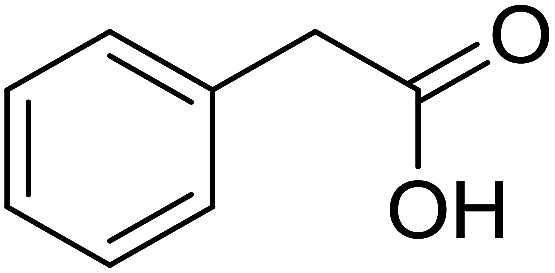	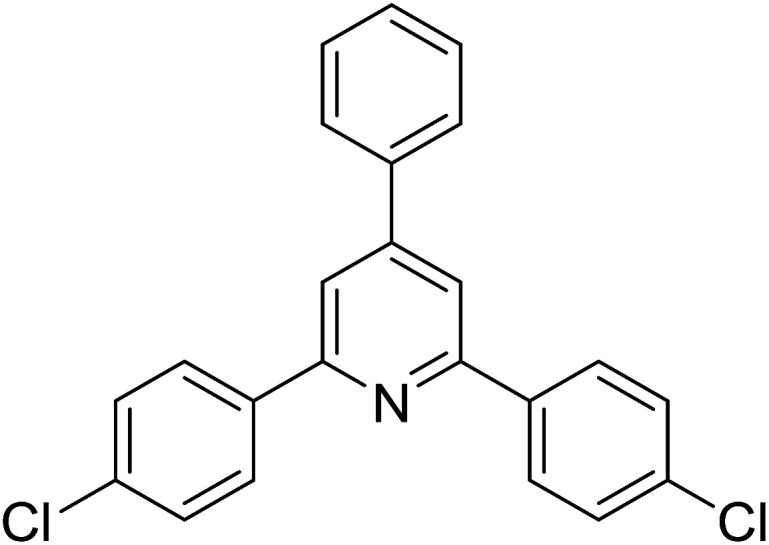	55
6	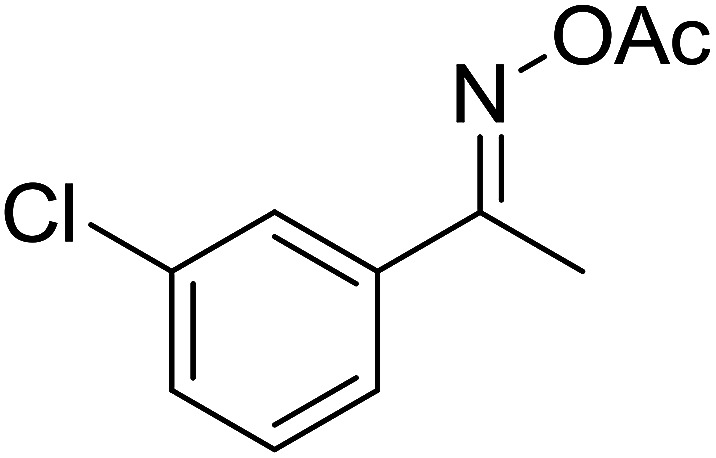	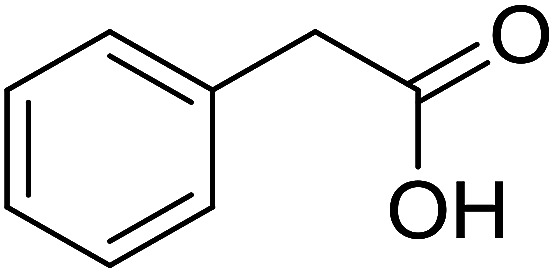	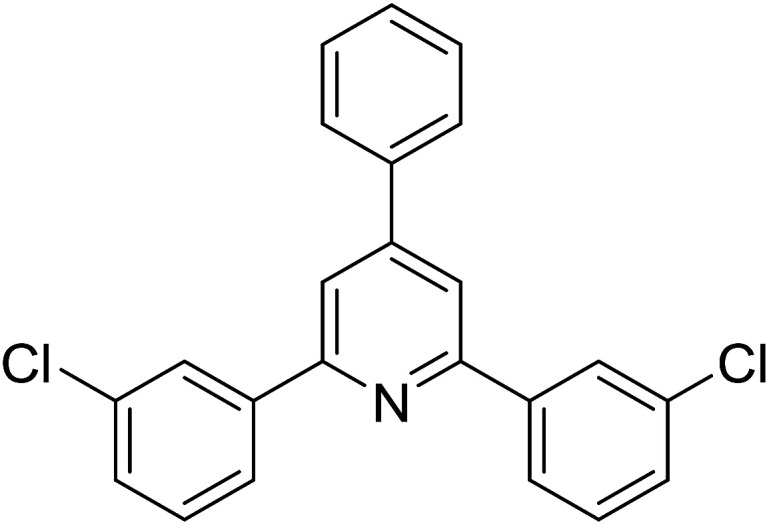	68
7	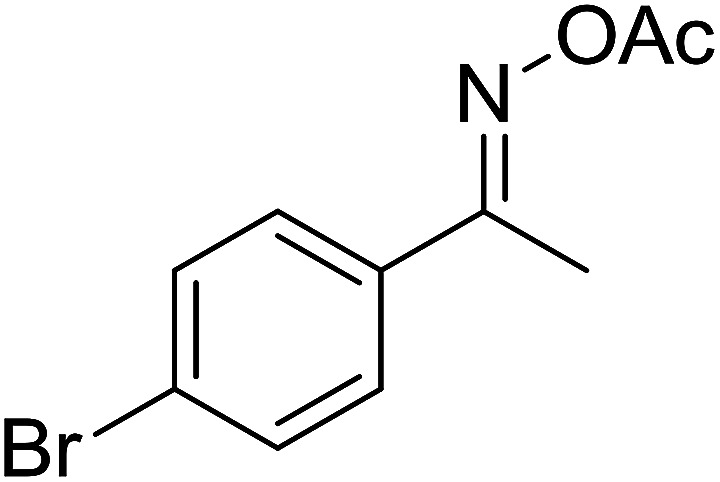	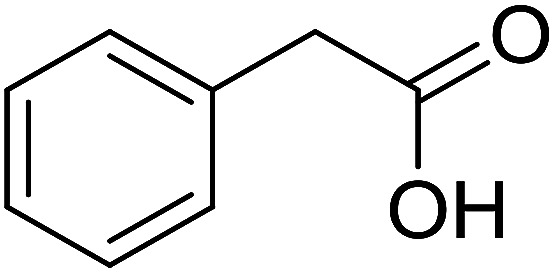	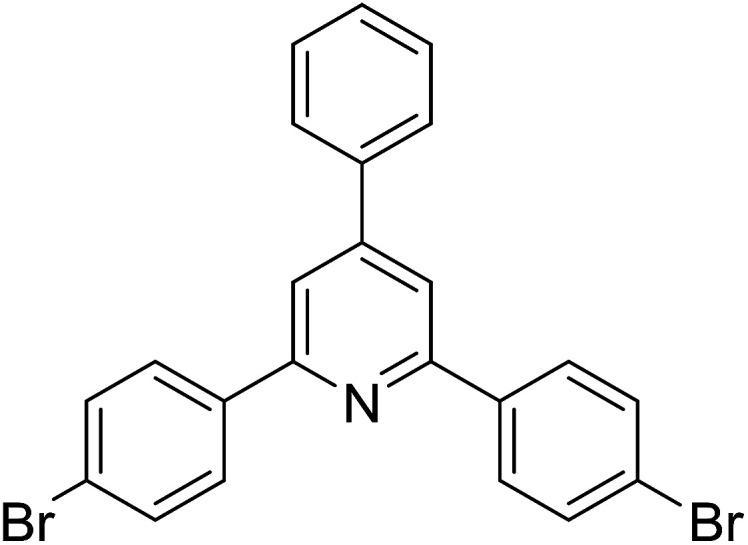	73
8	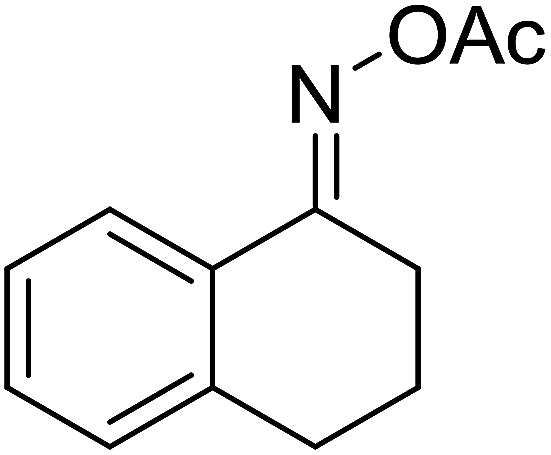	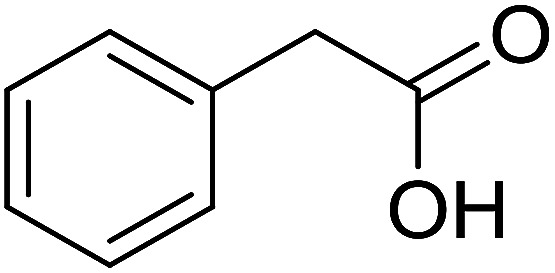	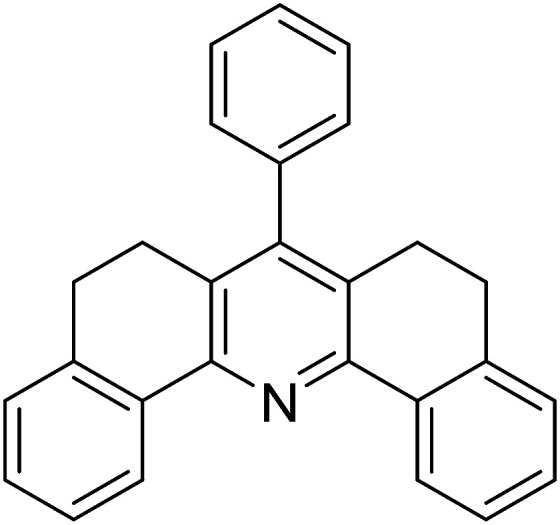	69
9	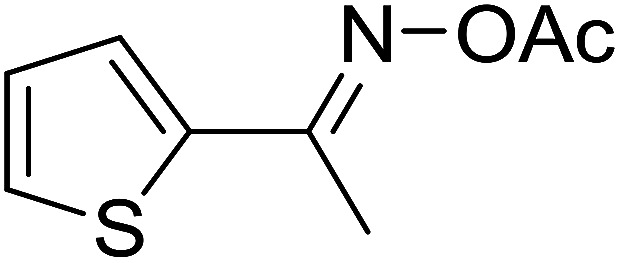	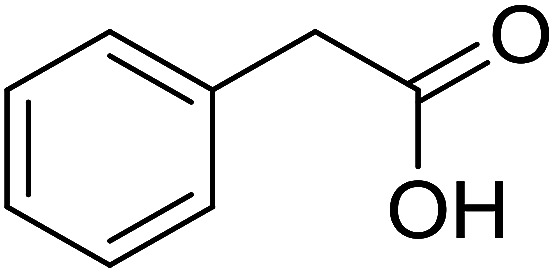	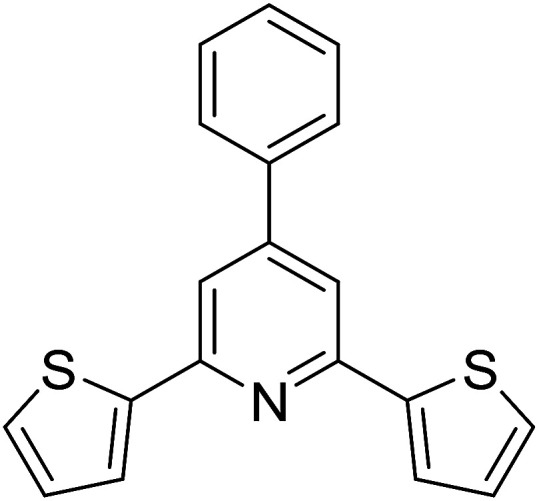	72
10	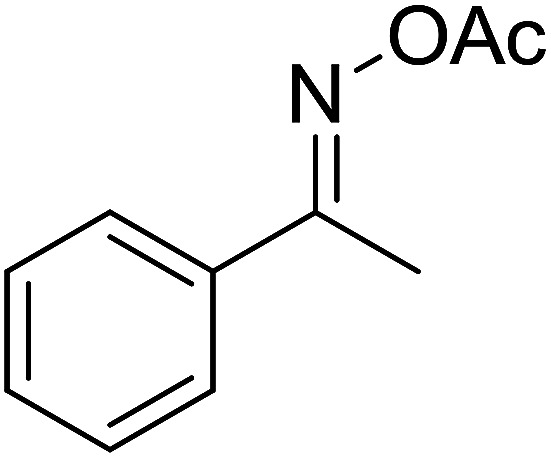	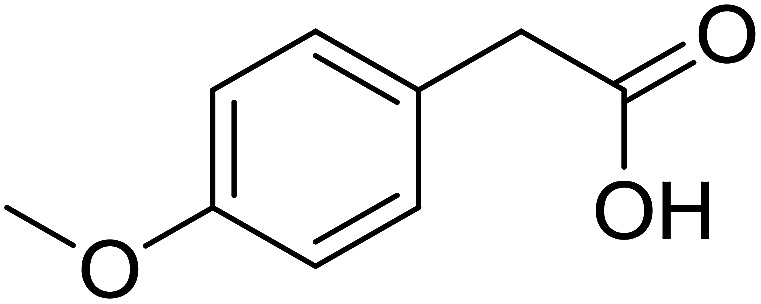	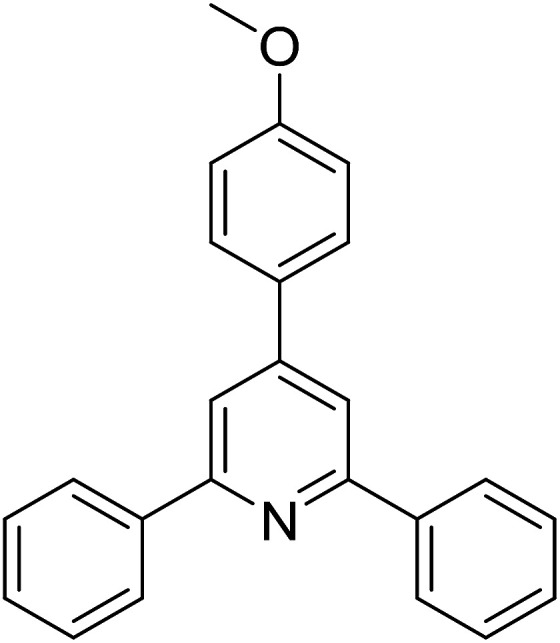	54
11	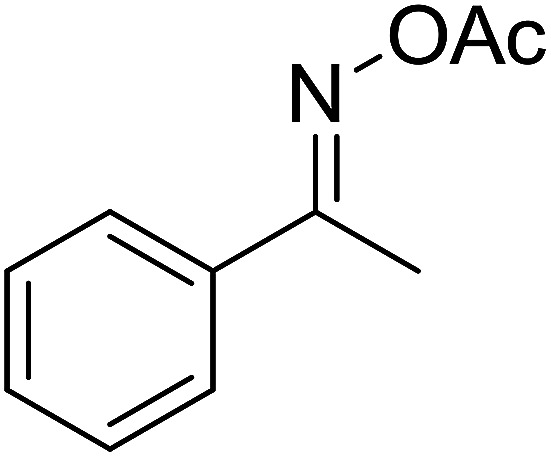	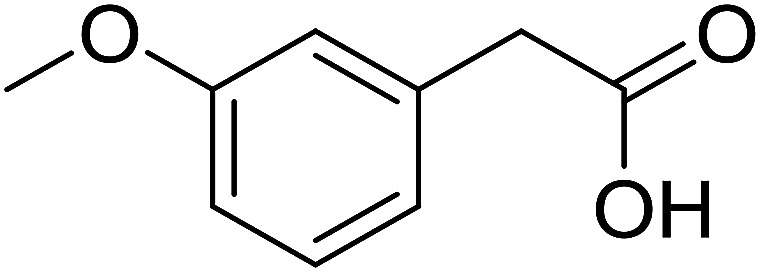	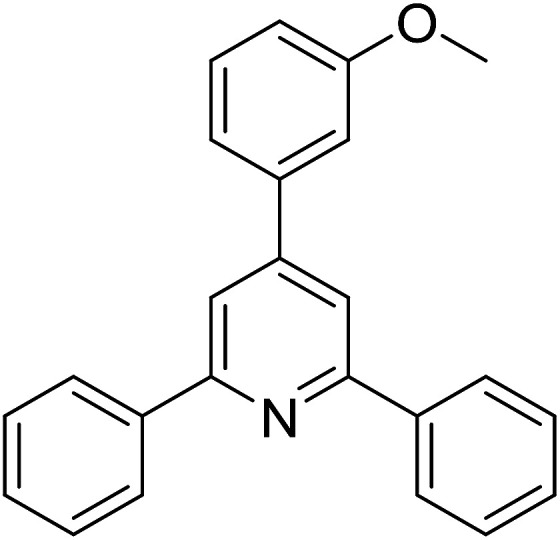	53
12	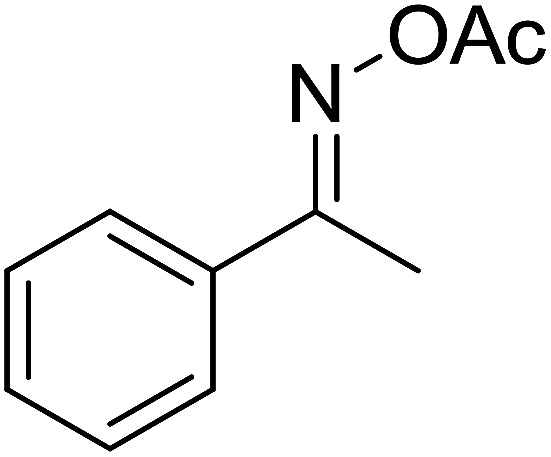	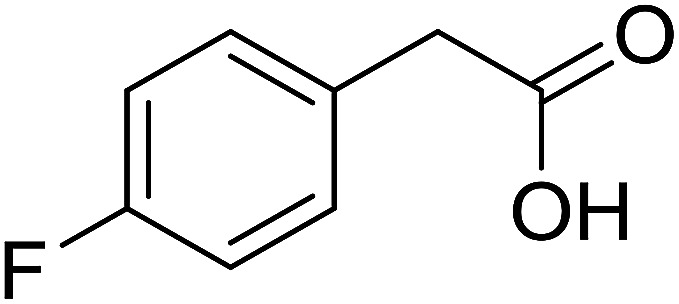	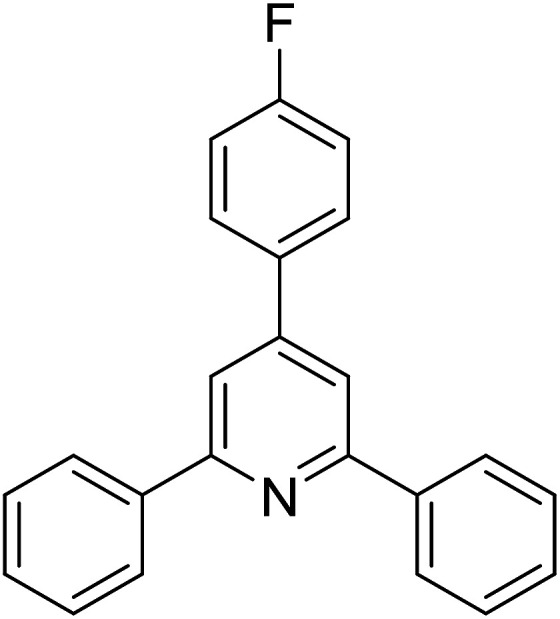	72
13	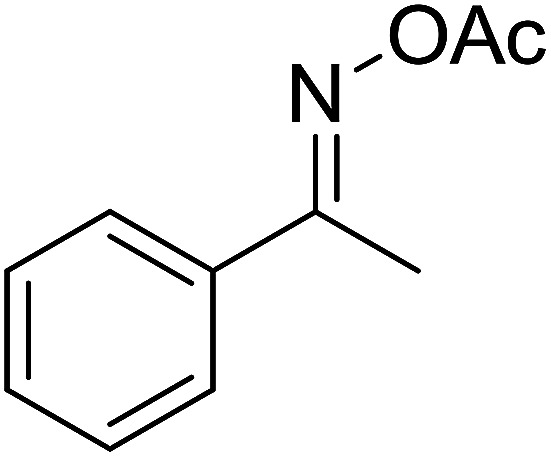	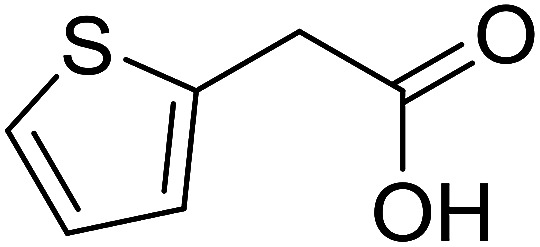	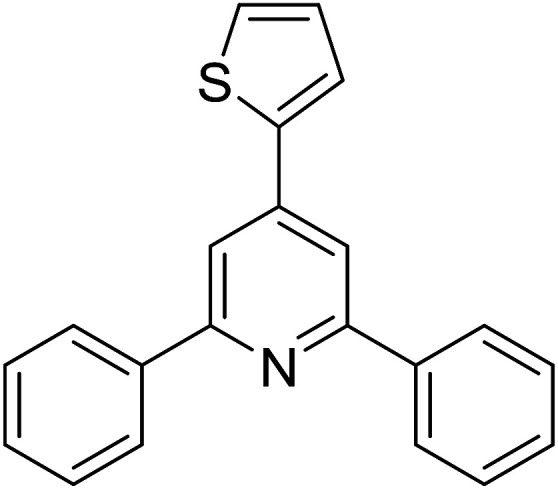	53
14	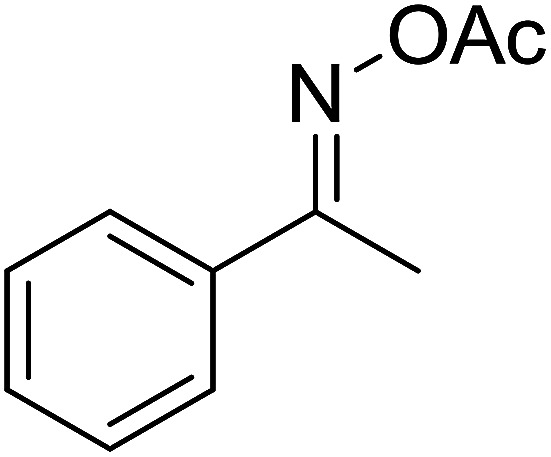	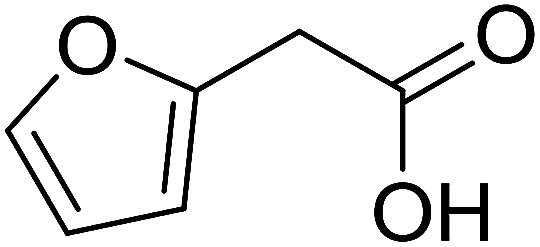	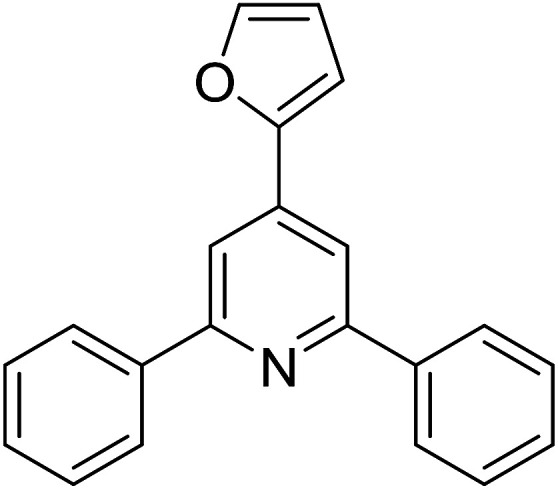	59
15	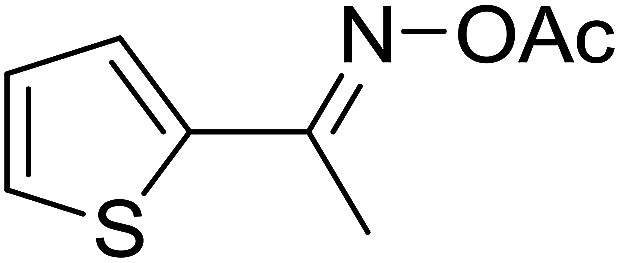	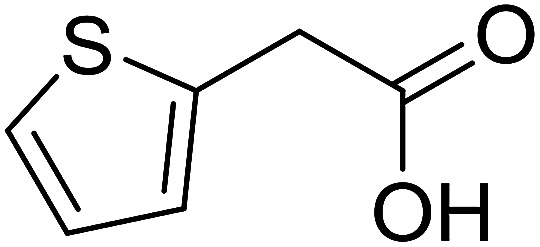	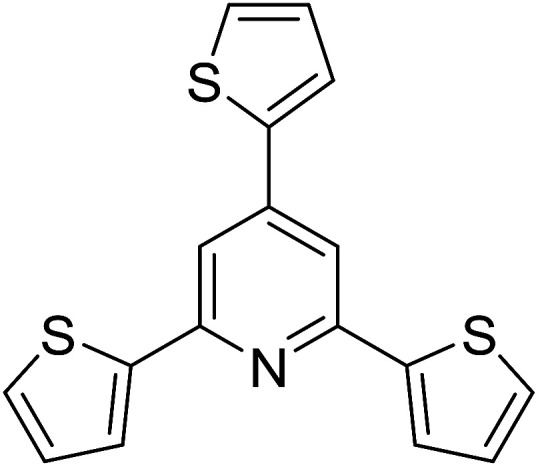	50

a Reaction conditions: phenylacetic acids (0.2 mmol), ketoximes (0.6 mmol), DTBP (0.6 mmol), toluene (2 mL), La_0.6_Sr_0.4_CoO_3_ catalyst (5 mol%), 8 h at 140 °C under air for 2 h and under argon for 6 h.

b Isolated yield.

## 4. Conclusions

In summary, a strontium-doped lanthanum cobaltite perovskite with the formula of La_0.6_Sr_0.4_CoO_3_ was prepared *via* a gelation and calcination method, and used as a heterogeneous catalyst for the synthesis of triphenylpyridines *via* a cyclization reaction between ketoximes and phenylacetic acids. The reaction proceeded *via* the oxidative functionalization of the sp^3^ C–H bond in phenylacetic acid in the presence of di-*tert*-butylperoxide as the oxidant. The La_0.6_Sr_0.4_CoO_3_-catalyzed transformation was significantly controlled by the solvent, and toluene was the best option. La_0.6_Sr_0.4_CoO_3_ was more active towards the oxidative cyclization reaction than numerous homogeneous and heterogeneous catalysts. Additionally, the strontium-doped perovskite offered dramatically higher catalytic activity than the pristine LaCoO_3_. Single oxides or salts of lanthanum, strontium, and cobalt demonstrated low catalytic performance for the transformation, indicating the importance of the synergistic effect in the perovskite catalyst. Furthermore, the La_0.6_Sr_0.4_CoO_3_ catalyst was facilely recovered and reused for the synthesis of triphenylpyridines without a considerable decline in its catalytic efficiency. The new combination of ketoximes with easily available phenylacetic acids *via* the oxidative functionalization of sp^3^ C–H bonds under recyclable perovskite-based catalysis conditions will make this method intriguing to the chemical and pharmaceutical industries.

## Conflicts of interest

There are no conflicts to declare.

## Supplementary Material

RA-009-C9RA04096J-s001
