# Supplementary material for: Synthesis of triphenylpyridines via an oxidative cyclization reaction using Sr-doped LaCoO3 perovskite as a recyclable heterogeneous catalyst
Source: RSC Adv. 2019 Aug 1;9(41):23876–87. doi: 10.1039/c9ra04096j (PMC9069450; doi:10.1039/c9ra04096j)
Supplement: RA-009-C9RA04096J-s001 [file RA-009-C9RA04096J-s001.pdf]

Electronic Supplementary Material (ESI) for RSC Advances.
This journal is © The Royal Society of Chemistry 2019

Synthesis of triphenylpyridines via oxidative cyclization reaction using Sr-doped $LaCoO_3$ perovskite as a recyclable heterogeneous catalyst

Thu N. M. Le, Son H. Doan, Phuc H. Pham, Khang H. Trinh, Tien V. Huynh, Tien T. T. Tran, Minh-Vien Le, Tung T. Nguyen*, Nam T. S. Phan*

Faculty of Chemical Engineering, HCMC University of Technology, VNU-HCM,

268 Ly Thuong Kiet, District 10, Ho Chi Minh City, Viet Nam

*Email: tungtn@hcmut.edu.vn, ptsnam@hcmut.edu.vn

Ph: (+84 8) 38647256 ext. 5681 Fx: (+84 8) 38637504

Supporting Information

Materials and instrumentation

All reagents and starting materials were obtained commercially from Sigma-Aldrich and Merck, and were used as received without any further purification unless otherwise noted. Nitrogen physisorption measurements were conducted using a Micromeritics 2020 volumetric adsorption analyzer system. Samples were pretreated by heating under vacuum at 150 ^{o}C for 3 h. X-ray powder diffraction (XRD) patterns were recorded using a Cu Kα radiation source on a D8 Advance Bruker powder diffractometer. Scanning electron microscopy studies were conducted on a S4800 Scanning Electron Microscope (SEM). Fourier transform infrared (FT-IR) spectra were obtained on a Nicolet 6700 instrument, with samples being dispersed on potassium bromide pallets.

Gas chromatographic (GC) analyses were performed using a Shimadzu GC 2010-Plus equipped with a flame ionization detector (FID) and an SPB-5 column (length = 30 m, inner diameter = 0.25 mm, and film thickness = 0.25 μm). The temperature program for GC analysis heated samples from 150 to 280 ^{o}C at 40 ^{o}C/min; held them at 280 ^{o}C for 12 min. Inlet and detector temperatures were set constant at 280 ^{o}C. The GC yield was calculated using diphenyl ether as the internal standard.

GC-MS analyses were analyzed on a Shimadzu GCMS-QP2010Ultra with a ZB-5MS column (length = 30 m, inner diameter = 0.25 mm, and film thickness = 0.25 μm). The temperature program for GC-MS analysis held samples at 50 ^{o}C for 2 min; heated samples from 50 to 280°C at 10 ^{o}C/min and held them at 280 ^{o}C for 10 min. Inlet temperature was set constant at 280 ^{o}C. MS spectra were compared with the spectra gathered in the NIST library. The ^{1}H NMR and ^{13}C NMR were recorded on Bruker AV 500 spectrometers using residual solvent peak as a reference.

Synthesis of strontium-doped lanthanum cobaltite perovskite catalyst

The strontium-doped lanthanum cobaltite perovskite powder was prepared via gelation and calcination approach. The nitrate salts of lanthanum ($La(NO_3)_3.6H_2O$, 3.90 g, 0.009 mol), strontium ($Sr(NO_3)_2$, 1.28 g, 0.006 mol) and cobalt ($Co(NO_3)_2.6H_2O$, 4.45 g, 0.015 mol) were dissolved in deionized water (100 mL) with vigorous stirring. Subsequently, citric acid (11.3 g, 0.06 mol) as chelating agent was added to the solution. The resulting

mixture was then heated at 80 °C under continuous stirring for 4 h in a water bath, and the clear pink solution was transformed into red gel. After the gelation step, the red gel was burnt at 140 °C overnight in an electric oven to produce soft-ash form. In order to obtain the perovskite, the ash was pre-calcined at 400 °C for 0.5 h, pulverized, and then calcined at 500 °C under air for 8 h. The resulting powder was consequently ball-milled in ethanol media using 5 mm diameter zirconia balls for 12 h at room temperature, and dried overnight in an electric oven at 140 °C to achieve the strontium-doped lanthanum cobaltite perovskite ($La_{0.6}Sr_{0.4}CoO_3$, 2.56 g).

General procedure for GC yields

In a representative experiment, a mixture of acetophenone oxime acetate (0.106 g, 0.6 mmol), phenylacetic acid (0.027 g, 0.2 mmol), and diphenyl ether (16 µL, 0.1 mmol) as an internal standard was added into a 12-mL screw-cap pressurized vial containing toluene (2 mL). The reaction mixture was magnetically stirred and heated for 5 min. The $La_{0.6}Sr_{0.4}CoO_3$ perovskite catalyst (2.3 mg, 5 mol%) was then added into the vial. The reaction mixture was magnetically stirred for 2 min to disperse entirely the catalyst in the liquid phase, followed by the addition of the di-*tert*-butylperoxide (DTBP, 0.14 mL, 0.6 mmol). The resulting mixture was stirred at 140 °C for 2 h. After that, the reactor tube was back-filled with argon and was subsequently stirred at 140 °C for 6 h. The reaction mixture was diluted with ethyl acetate (5 mL) and washed with saturated $NaHCO_3$ solution (5 mL). The organic layer was dried by anhydrous Na_2SO_4. Reaction yields were recorded from GC analysis results concerning the diphenyl ether internal standard.

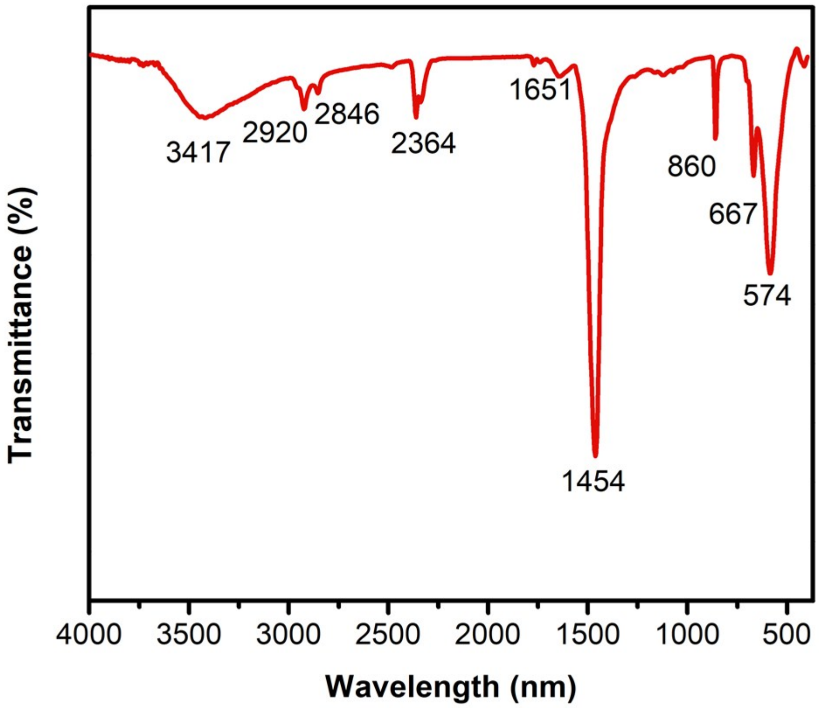

Fig. S1. FT-IR result of the $La_{0.6}Sr_{0.4}CoO_3$ perovskite catalyst.

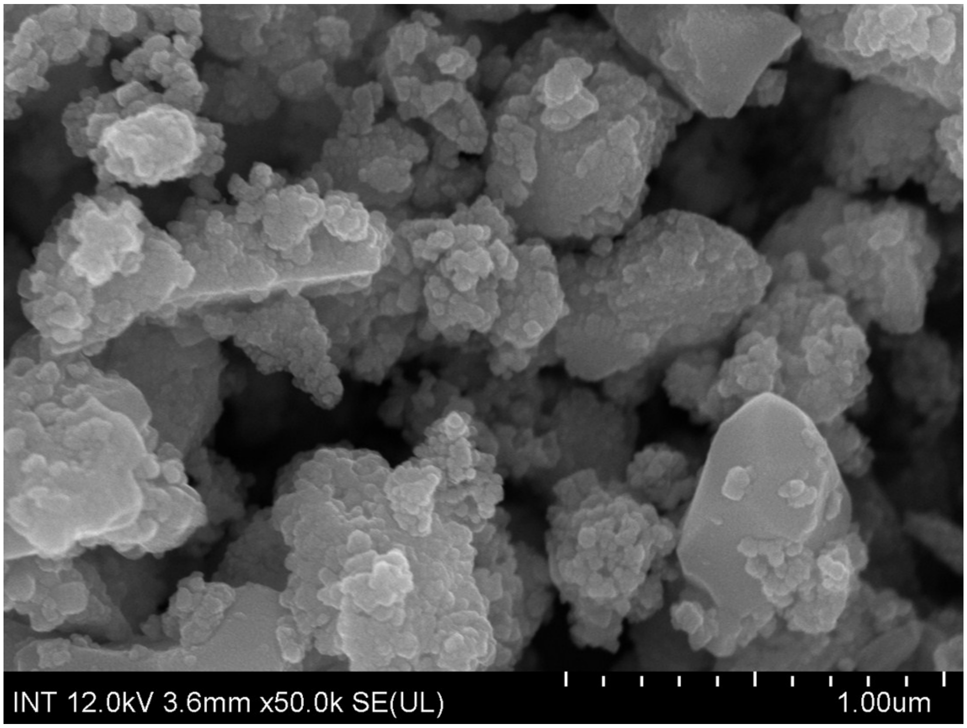

Fig. S2. SEM micrograph of the $La_{0.6}Sr_{0.4}CoO_3$ perovskite catalyst.

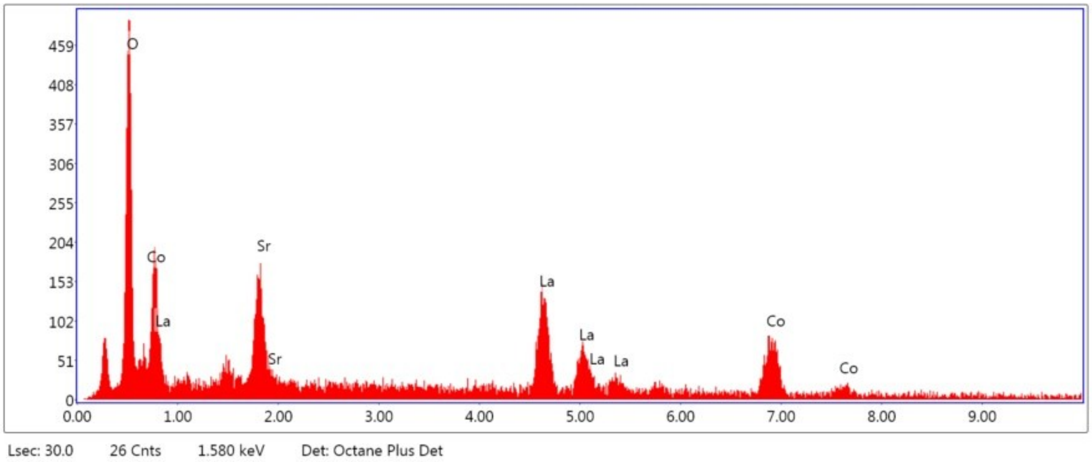

Fig. S3. EDX patterns of the $La_{0.6}Sr_{0.4}CoO_3$ perovskite catalyst.

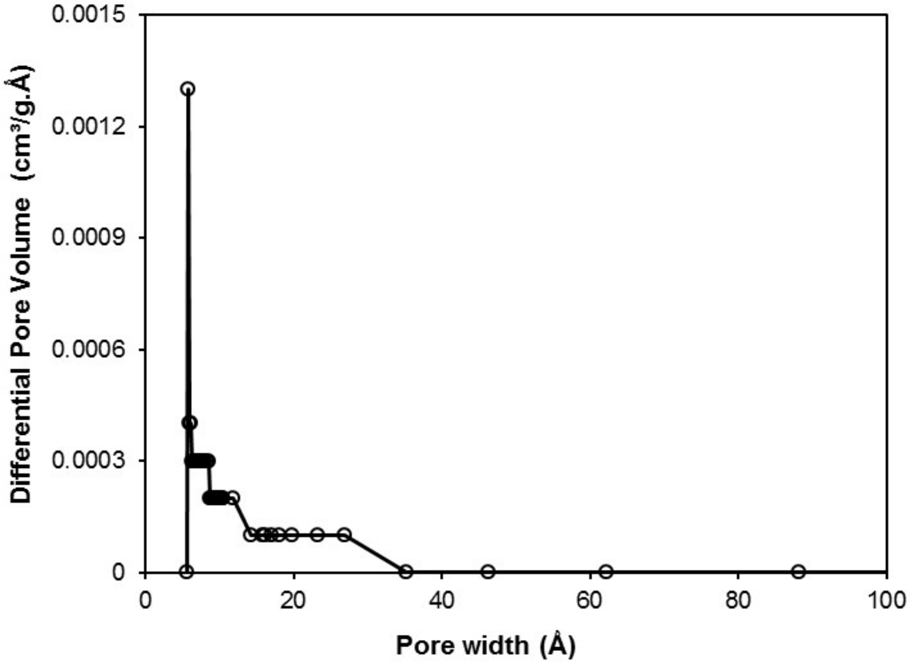

Fig. S4. Pore size distribution of the $La_{0.6}Sr_{0.4}CoO_3$ perovskite catalyst.

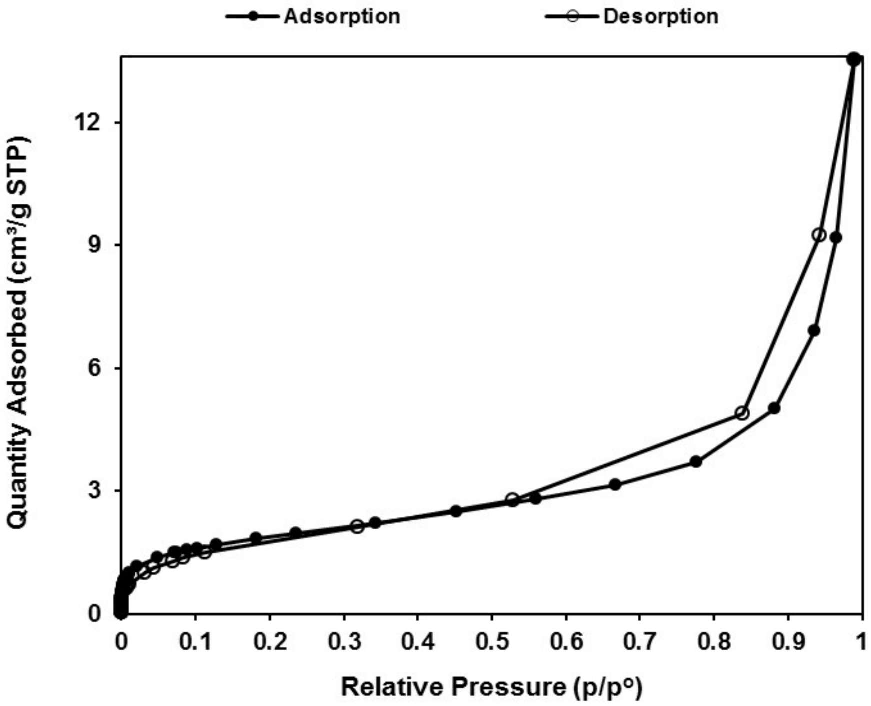

Fig. S5. Nitrogen adsorption/desorption isotherm of the $La_{0.6}Sr_{0.4}CoO_3$ perovskite.

Adsorption data are shown as closed triangles and desorption data as open triangles.

Table 1. Optimization of reaction conditions[a].

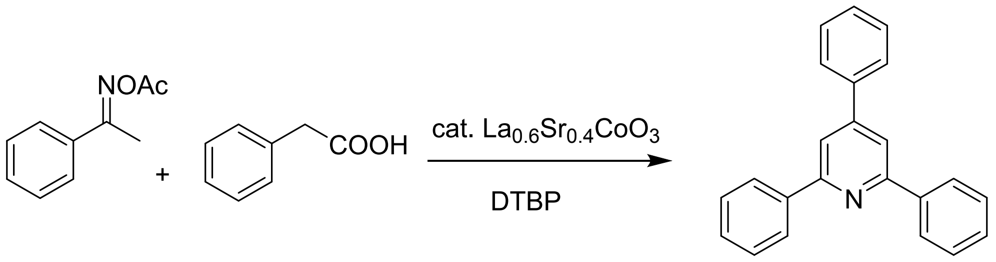

Entry	solvent	temperature (°C)	ketoxime amount (equiv.)	catalyst amount (mol%)	oxidant	oxidant amount (equiv.)	yield[b] (%)
1	PhCl	140	3	5	DTBP	3	54
2	NMP	140	3	5	DTBP	3	1
3	Heptane	140	3	5	DTBP	3	22
4	DCB	140	3	5	DTBP	3	12
5	DMSO	140	3	5	DTBP	3	3
6	Diglyme	140	3	5	DTBP	3	2
7	THF	140	3	5	DTBP	3	15
8	Dioxane	140	3	5	DTBP	3	2
9	DMF	140	3	5	DTBP	3	1
10	DMAc	140	3	5	DTBP	3	1
11	Toluene	140	3	5	DTBP	3	83
12	Toluene	RT	3	5	DTBP	3	0
13	Toluene	80	3	5	DTBP	3	0

14	Toluene	100	3	5	DTBP	3	0
15	Toluene	120	3	5	DTBP	3	17
16	Toluene	140	3	5	DTBP	3	83
17	Toluene	140	1	5	DTBP	3	10
18	Toluene	140	2	5	DTBP	3	18
19	Toluene	140	3	5	DTBP	3	83
20	Toluene	140	4	5	DTBP	3	83
21	Toluene	140	3	0	DTBP	3	4
22	Toluene	140	3	1	DTBP	3	21
23	Toluene	140	3	3	DTBP	3	51
24	Toluene	140	3	5	DTBP	3	83
25	Toluene	140	3	7	DTBP	3	87
26	Toluene	140	3	5	$K_2S_2O_8$	3	8
27	Toluene	140	3	5	$AgNO_3$	3	11
28	Toluene	140	3	5	Oxygen	3	2
29	Toluene	140	3	5	H_2O_2	3	3
30	Toluene	140	3	5	aqTBHP	3	55
31	Toluene	140	3	5	dcTBHP	3	60
32	Toluene	140	3	5	CHP	3	7
33	Toluene	140	3	5	DTBP	3	83

34	Toluene	140	3	5	DTBP	0	0
35	Toluene	140	3	5	DTBP	1	33
36	Toluene	140	3	5	DTBP	2	61
37	Toluene	140	3	5	DTBP	3	83
38	Toluene	140	3	5	DTBP	4	82

[a] Reaction conditions: Phenylacetic acid (0.2 mmol); solvent (2 mL); under air for 2 h and under argon for 6 h. [b] GC yield. DTBP = di-*tert*-butylperoxide, aqTBHP = *tert*-butyl hydroperoxide in water, dcTBHP = *tert*-butyl hydroperoxide in decane, CHP = cumyl hydroperoxide, NMP = *N*-methyl-2-pyrrolidone, DCB = 1,2-dichlorobenzene, DMSO = dimethylsulfoxide, DMF = *N*,*N*-dimethylformamide, DMAc = *N*,*N*-dimethylacetamide.

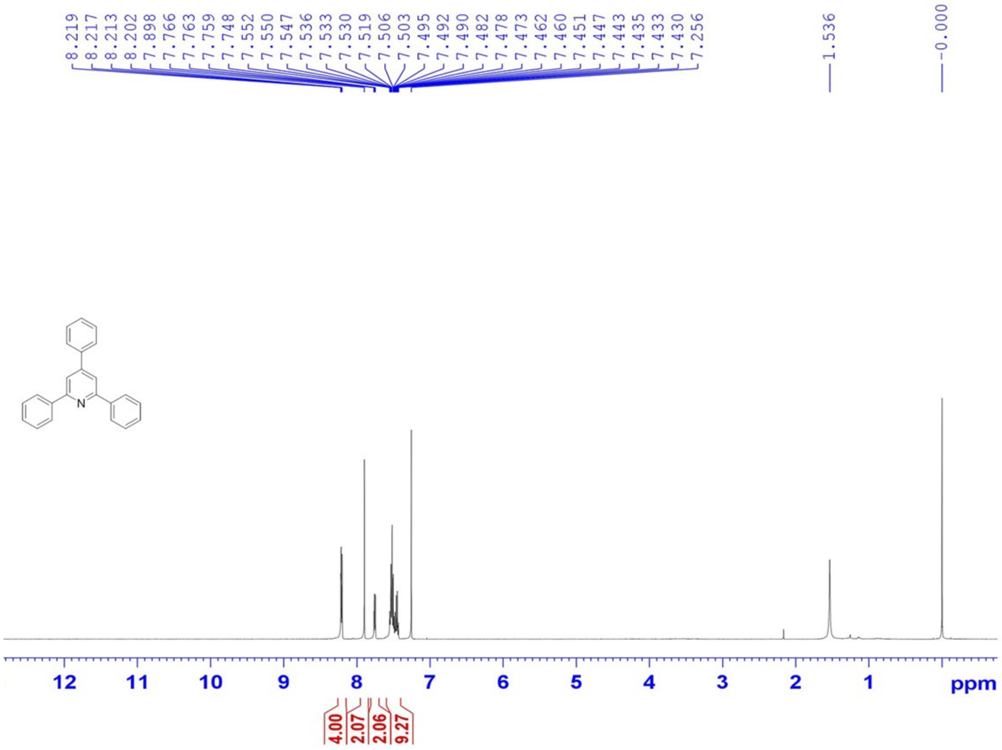

Fig.S6. ^{1}H-NMR spectra of 2,4,6-triphenylpyridine.

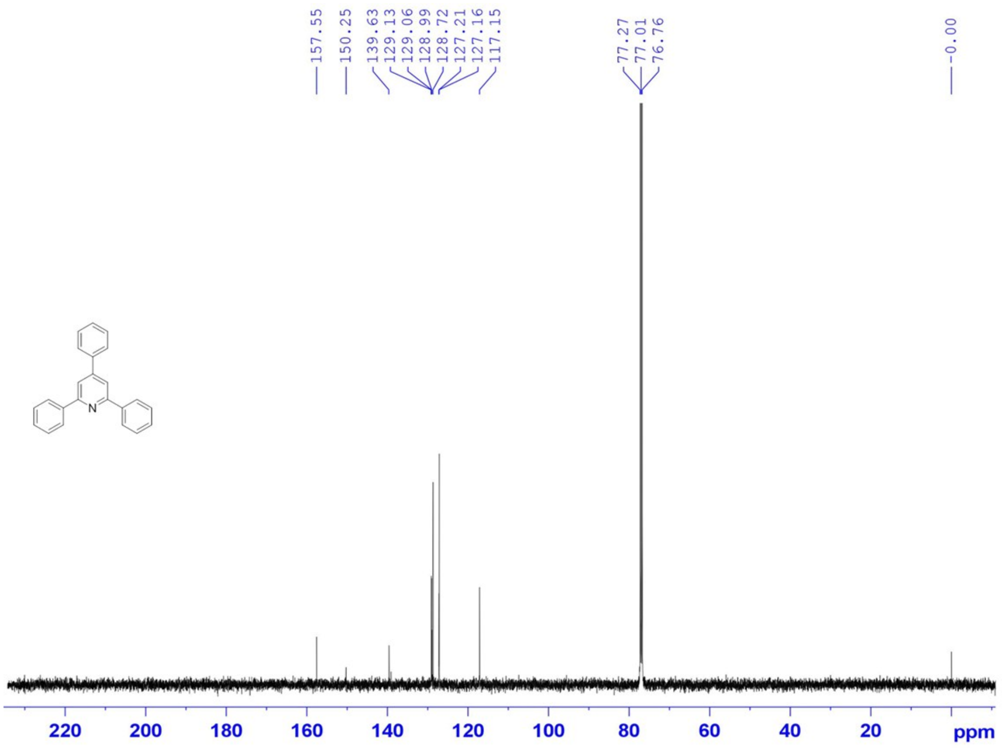

Fig.S7. ^{13}C-NMR spectra of 2,4,6-triphenylpyridine.

Characterization data for 2,4,6-triphenylpyridine

Prepared as shown in the general experimental procedure and purified on silica gel (230-400 mesh or 37-63 μm, ethyl acetate/hexane = 1:1 (v./v.), TLC silica gel 60 F_{254}, R_f = 0.40): White solid, 79% yield (48.5 mg). ^{1}H-NMR (500 MHz, $CDCl_3$) δ(ppm) 7.43 – 7.54 (m, 9H), 7.74 (d, *J* = 7.5 Hz, 2H), 7.89 (s, 2H), 8.20 (d, *J* = 7.5 Hz, 4H). ^{13}C NMR ($CDCl_3$, 125 MHz) δ(ppm) 117.1, 127.1, 127.2, 128.7, 128.9, 129.0, 129.1, 139.6, 150.2, 157.5.

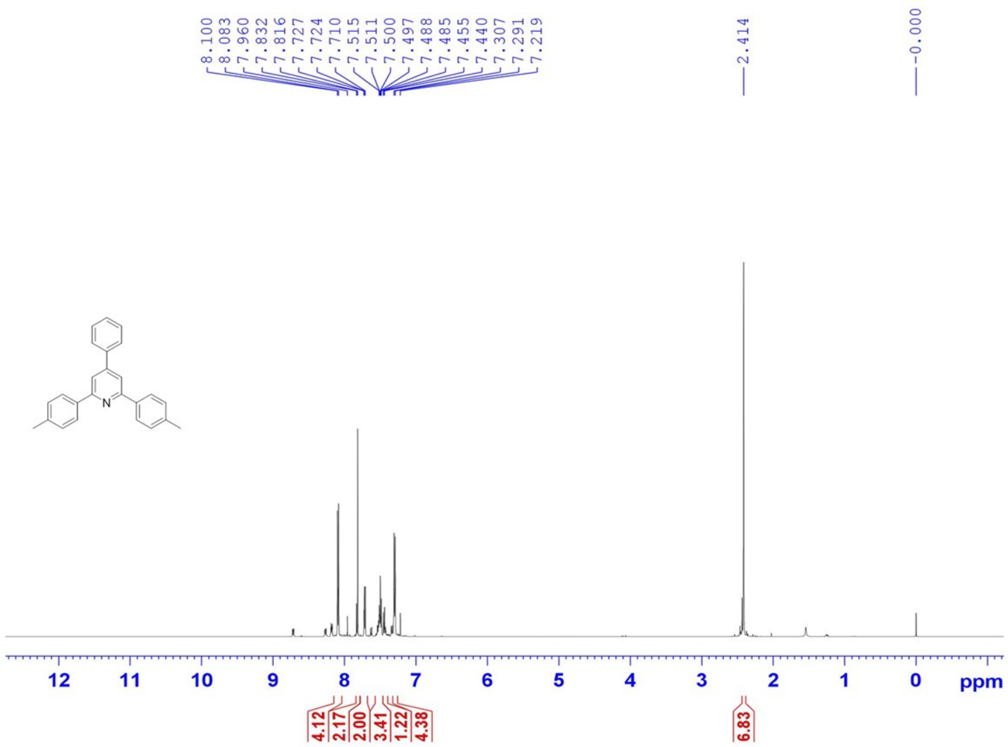

Fig.S8. ^{1}H-NMR spectra of 4-phenyl-2,6-di-*p*-tolylpyridine.

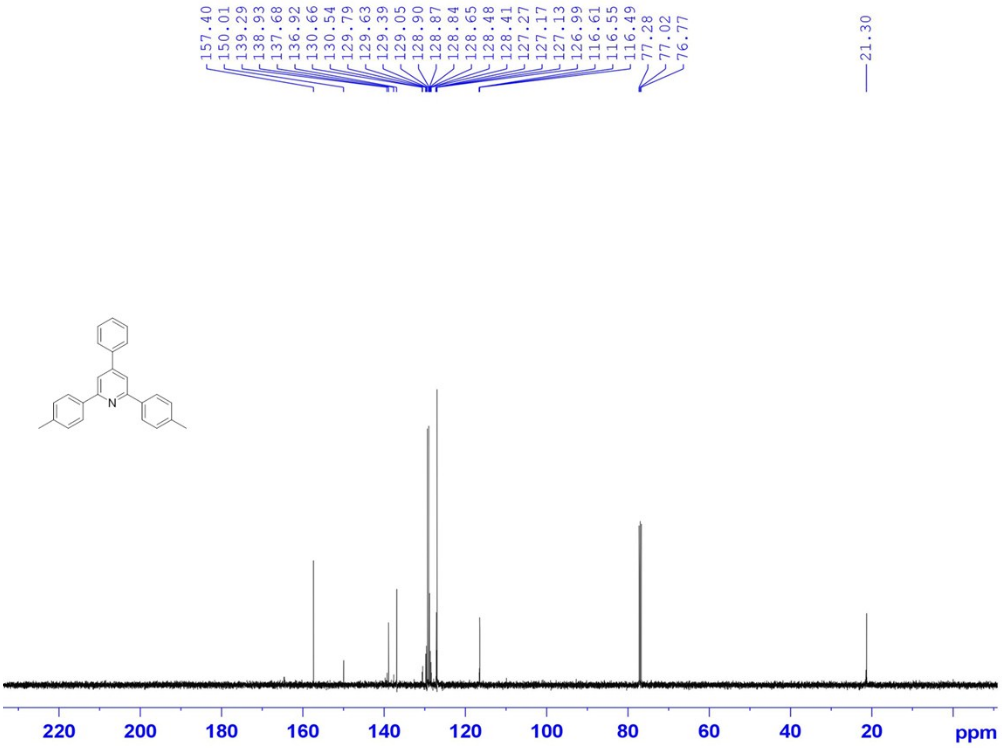

Fig.S9. ^{13}C-NMR spectra of 4-phenyl-2,6-di-*p*-tolylpyridine.

Characterization data for 4-phenyl-2,6-di-*p*-tolylpyridine

Prepared as shown in the general experimental procedure and purified on silica gel (230-400 mesh or 37-63 μm, ethyl acetate/hexane = 1:20 (v./v.), TLC silica gel 60 F_{254}, R_f = 0.35): Yellow solid, 62% yield (41.5 mg). ^{1}H-NMR (500 MHz, $CDCl_3$) δ(ppm) 2.41 (s, 6H), 7.29 (d, *J* = 8.0 Hz, 4H), 7.44 – 7.45 (m, 1H), 7.48 – 7.51 (m, 3H), 7.71 (d, *J* = 8.0 Hz, 2H), 7.81 (s, 2H), 8.08 (d, *J* = 8.0 Hz, 4H). ^{13}C NMR ($CDCl_3$, 125 MHz) δ(ppm) 21.3, 116.4, 116.5, 116.6, 127.1, 127.2, 128.4, 128.6, 128.8, 128.9, 129.0, 129.3, 129.6, 129.7, 130.5, 130.6, 136.9, 137.6, 138.9, 139.2, 150.0, 157.4.

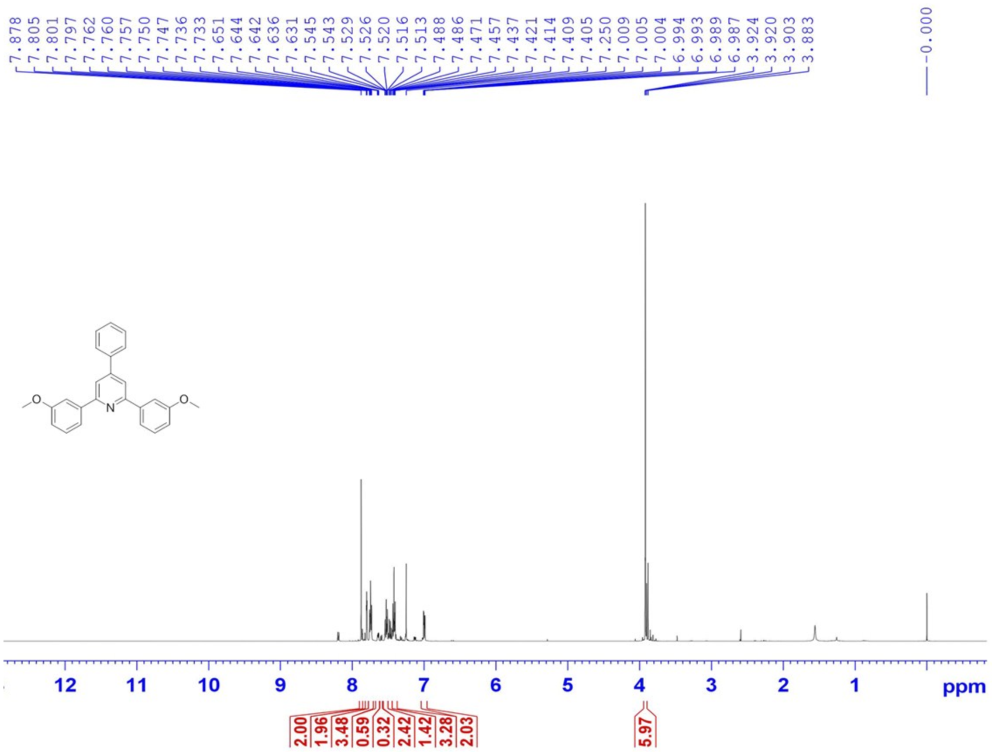

Fig.S10. ¹H-NMR spectra of 2,6-bis(3-methoxyphenyl)-4-phenylpyridine.

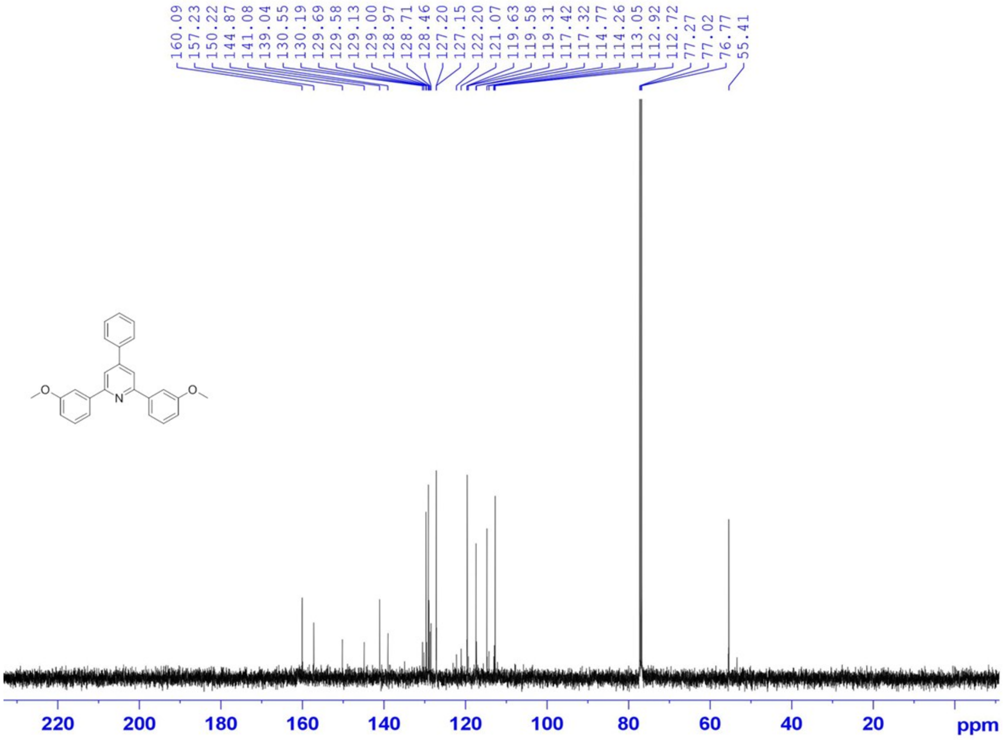

Fig.S11. ^{13}C-NMR spectra of 2,6-bis(3-methoxyphenyl)-4-phenylpyridine.

Characterization data for 2,6-bis(3-methoxyphenyl)-4-phenylpyridine

Prepared as shown in the general experimental procedure and purified on silica gel (230-400 mesh or 37-63 μm, ethyl acetate/hexane = 1:15 (v./v.), TLC silica gel 60 F_{254}, R_f = 0.4): Yellow solid, 74% yield (54.3 mg). ^{1}H-NMR (500 MHz, $CDCl_3$) δ(ppm) 3.92 (s, 6 H), 6.98 (ddd, *J* = 8.0 Hz, 2.5 Hz, 0.5 Hz, 2H), 7.40-7.43 (m, 3H), 7.45-7.48 (m, 1H), 7.51-7.54 (m, 2H), 7.73-7.76 (m, 3H), 7.79 (t, *J* = 2.0 Hz, 2H), 7.87 (s, 2H). ^{13}C NMR (CDCl3, 125 MHz) δ(ppm) 55.4, 112.7, 112.9, 114.7, 117.4, 119.5, 127.2, 128.7, 129.0, 129.5, 129.6, 130.5, 139.0, 141.0, 144.8, 150.2, 157.2, 160.0.

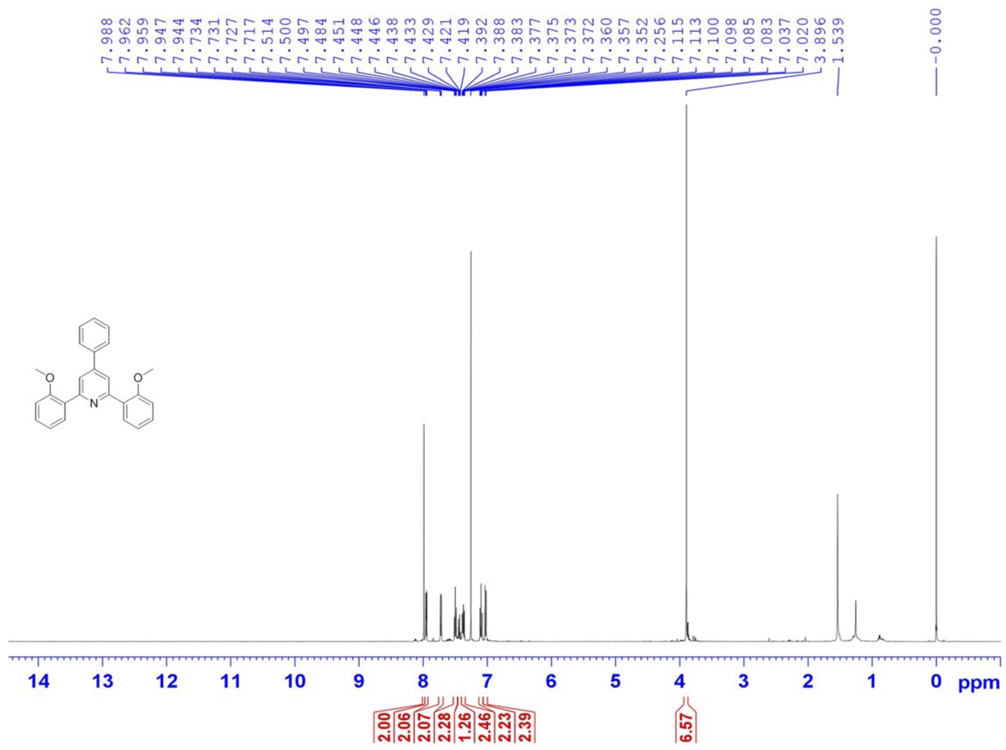

Fig.S12. ¹H-NMR spectra of 2,6-bis(2-methoxyphenyl)-4-phenylpyridine.

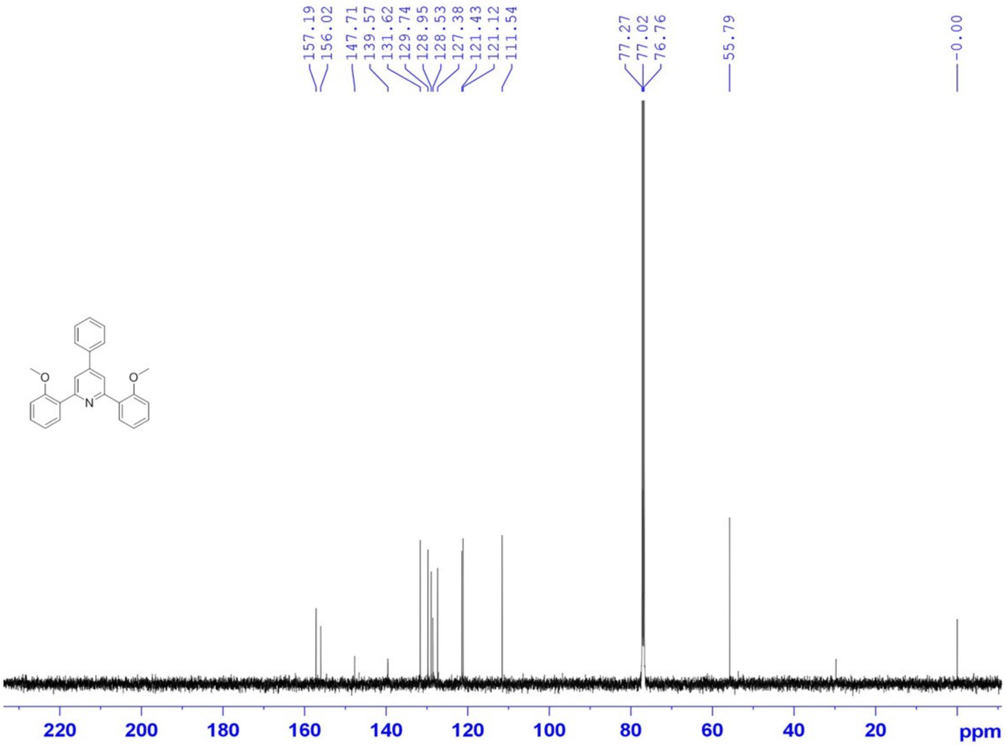

Fig.S13. ^{13}C-NMR spectra of 2,6-bis(2-methoxyphenyl)-4-phenylpyridine.

Characterization data for 2,6-bis(2-methoxyphenyl)-4-phenylpyridine

Prepared as shown in the general experimental procedure and purified on silica gel (230-400 mesh or 37-63 μm, ethyl acetate/hexane = 1:20 (v./v.), TLC silica gel 60 F_{254}, R_f = 0.4): Yellow solid, 67 % yield (49.2 mg). ^1H-NMR (500 MHz, $CDCl_3$) δ(ppm) 3.89 (s, 6H). 7.02 (d, *J* = 8.0 Hz, 2H), 7.08 (td, *J* = 7.5 Hz, 1.0 Hz, 2H), 7. 35 – 7.39 (m, 2H), 7.41 (tt, *J* = 8.0 Hz, 1.5 Hz, 1H), 7.48-7.51 (m, 2H), 7.71 – 7.73 (m, 2H), 7.94 (dd, *J* = 7.5 Hz, 1.5 Hz, 2H), 7.98 (s, 2H). ^{13}C NMR ($CDCl_3$, 125 MHz) δ(ppm) 55.7, 111.5, 121.1, 121.4, 127.3, 128.5, 128.9, 129.7, 131.6, 139.5, 147.7, 156.0, 157.1.

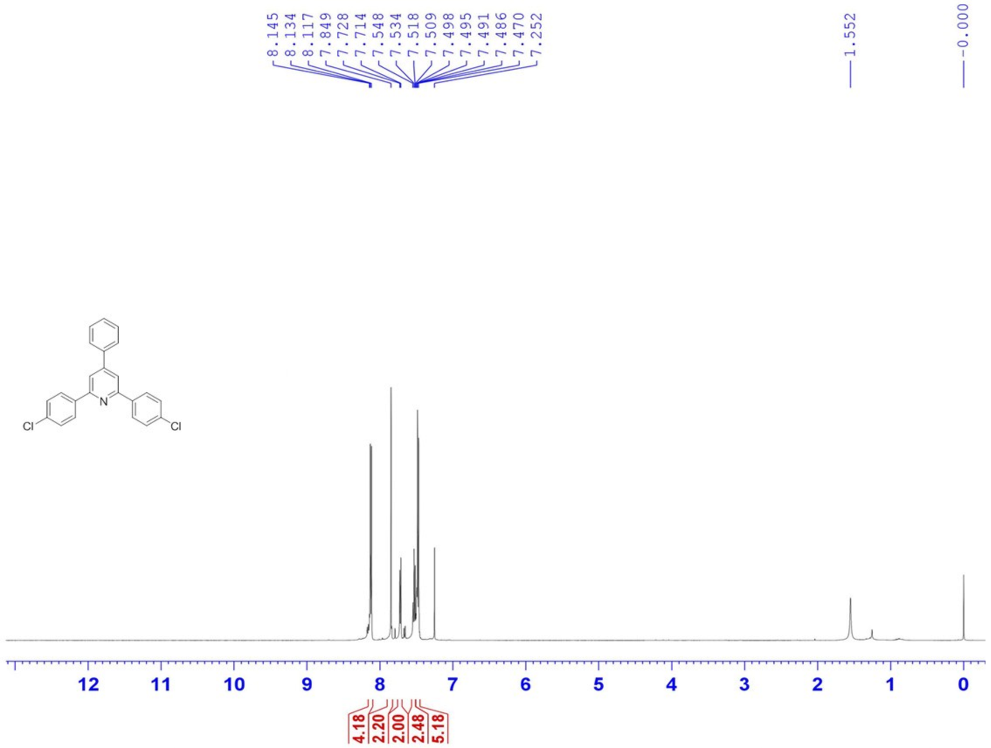

Fig.S14. ^{1}H-NMR spectra of 2,6-bis(4-chlorophenyl)-4-phenylpyridine.

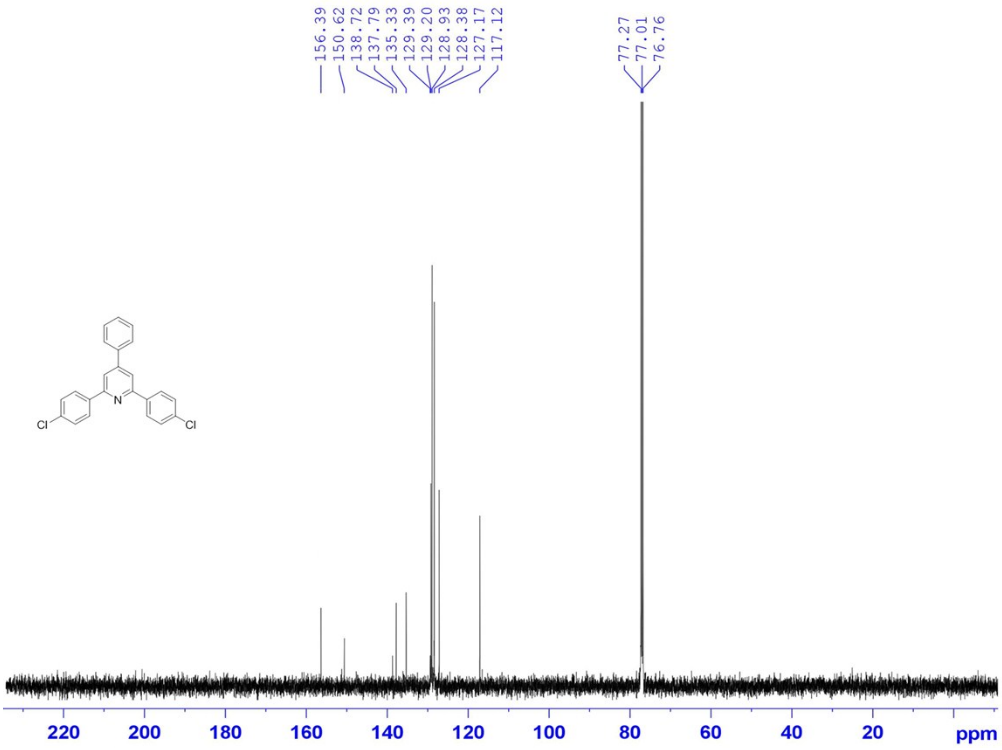

Fig.S15. ^{13}C-NMR spectra of 2,6-bis(4-chlorophenyl)-4-phenylpyridine.

Characterization data for 2,6-bis(4-chlorophenyl)-4-phenylpyridine

Prepared as shown in the general experimental procedure and purified on silica gel (230-400 mesh or 37-63 μm, ethyl acetate/hexane = 1:20 (v./v.), TLC silica gel 60 F_{254}, R_f = 0.35): Yellow solid, 55% yield (41.2 mg). ^{1}H-NMR (500 MHz, $CDCl_3$) δ(ppm) 7.47 – 7.49 (m, 5H), 7.50-7.54 (m, 2H), 7.71 (d, *J* = 7.0 Hz, 2H), 7.84 (s, 2H), 8.11 (d, *J* = 8.5 Hz, 4H).^{13}C NMR ($CDCl_3$, 125 MHz) δ(ppm) 117.1, 127.1, 128.3, 128.9, 129.2, 129.3, 135.3, 137.7, 138.7, 150.6, 156.3.

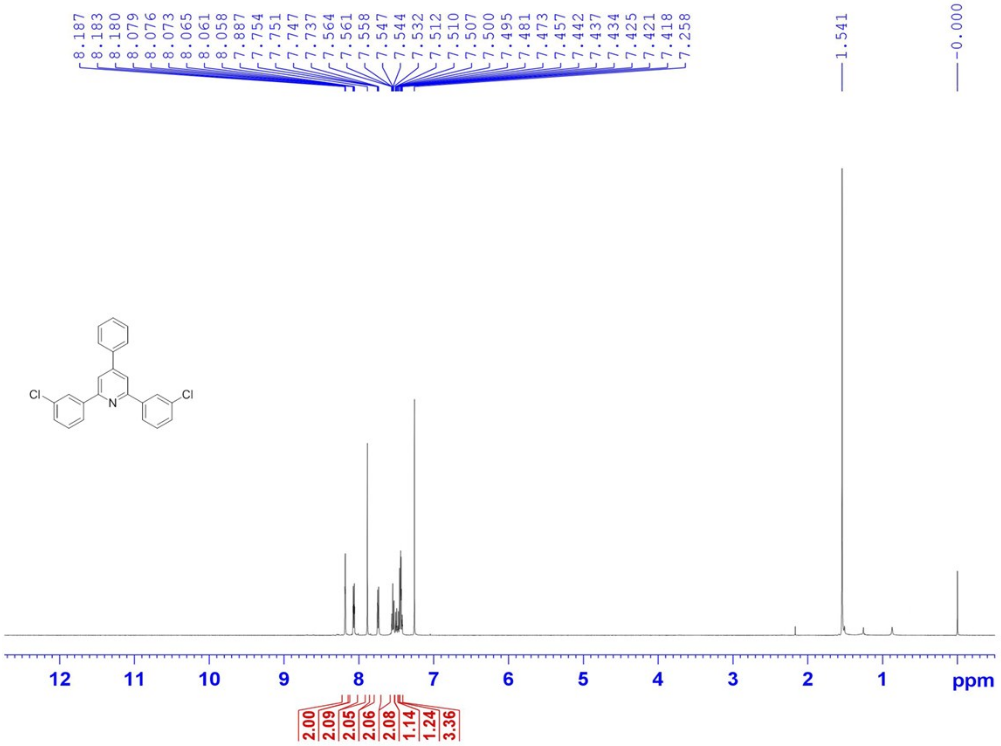

Fig.S16. ^{1}H-NMR spectra of 2,6-bis(3-chlorophenyl)-4-phenylpyridine.

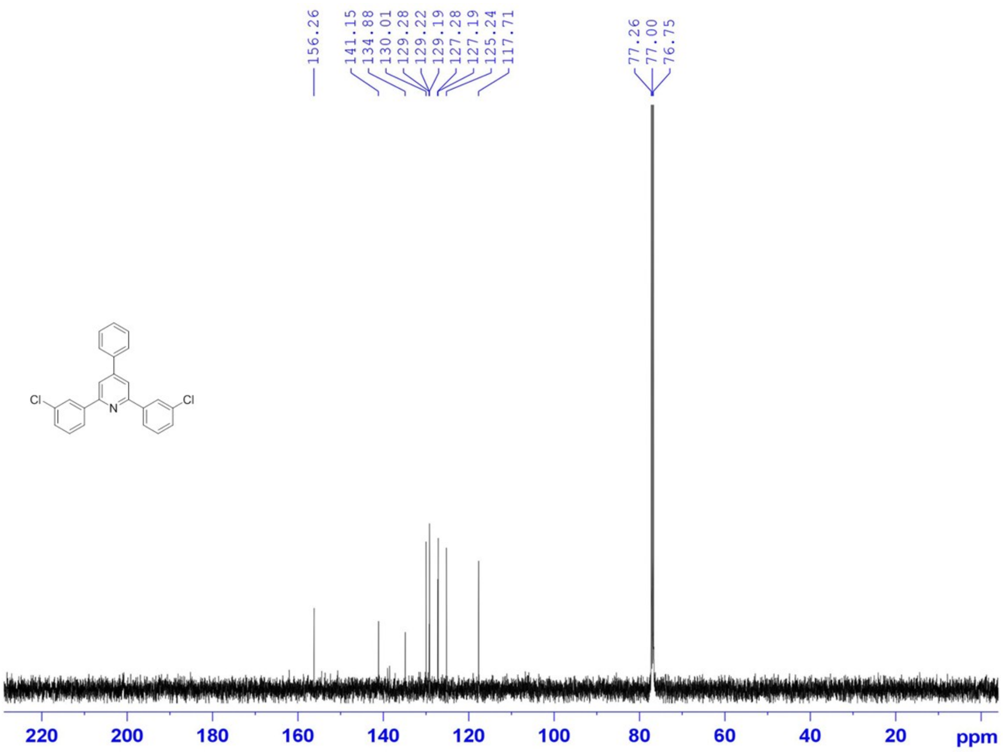

Fig.S17. ^{13}C-NMR spectra of 2,6-bis(3-chlorophenyl)-4-phenylpyridine.

Characterization data for 2,6-bis(3-chlorophenyl)-4-phenylpyridine

Prepared as shown in the general experimental procedure and purified on silica gel (230-400 mesh or 37-63 μm, ethyl acetate/hexane = 1:15 (v./v.), TLC silica gel 60 F_{254}, R_f = 0.4): Yellow solid, 68% yield (51 mg). ^{1}H-NMR (500 MHz, $CDCl_3$) δ(ppm) 7.41 – 7.51 (m, 5H), 7.53 (tt, J = 7.5 Hz, 1.5 Hz, 2H), 7.73 (dt, J = 7.5 Hz, 1.5 Hz, 2H), 7.88 (s, 2H), 8.05 (dt, J = 7.5 Hz, 1.5 Hz, 2 H), 8.18 (t, J = 1.5 Hz, 2H). ^{13}C NMR ($CDCl_3$, 125 MHz) δ(ppm) 117.7, 125.2, 127.1, 127.2, 129.1, 129.2, 129.2, 130.0, 134.8, 141.1, 156.2.

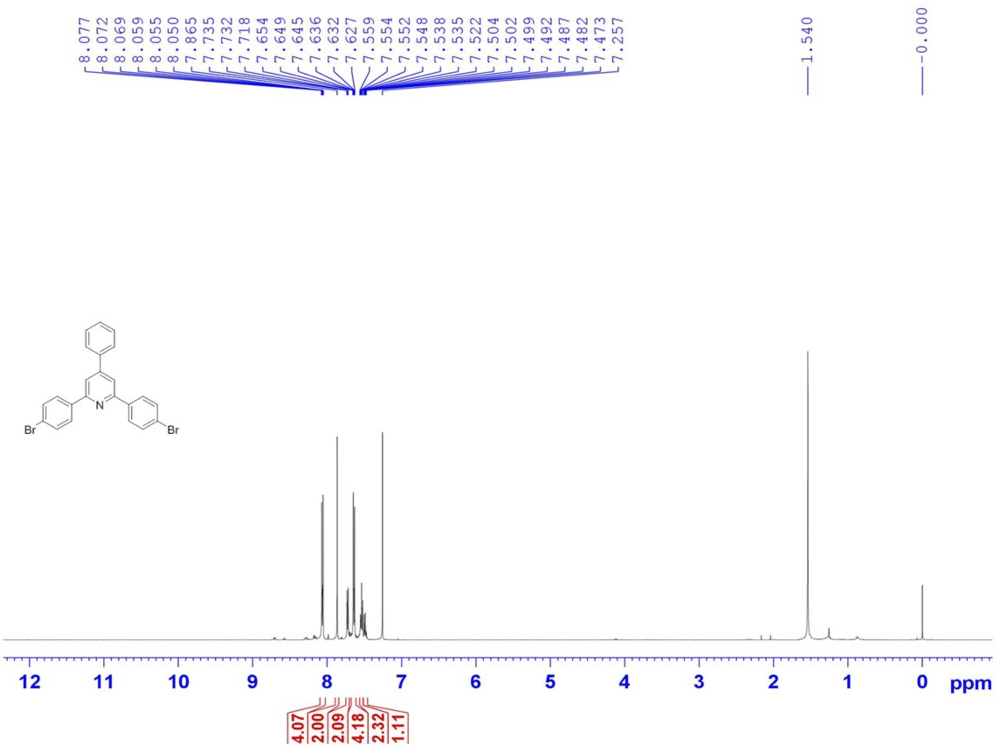

Fig.S18. ^{1}H-NMR spectra of 2,6-bis(4-bromophenyl)-4-phenylpyridine.

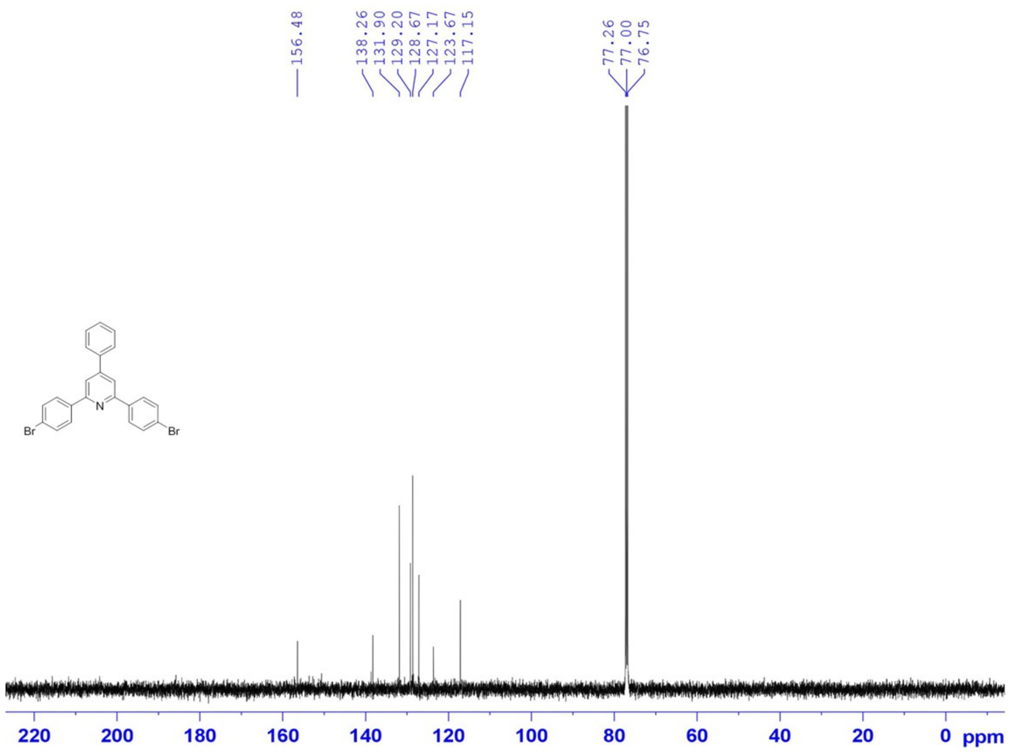

Fig.S19. ^{13}C-NMR spectra of 2,6-bis(4-bromophenyl)-4-phenylpyridine.

Characterization data for 2,6-bis(4-bromophenyl)-4-phenylpyridine

Prepared as shown in the general experimental procedure and purified on silica gel (230-400 mesh or 37-63 μm, ethyl acetate/hexane = 1:20 (v./v.), TLC silica gel 60 F_{254}, R_f = 0.35): Yellow solid, 73% yield (67.4 mg). ^{1}H-NMR (500 MHz, $CDCl_3$) δ(ppm) 7.47–7.55 (m, 3H), 7.62 (dt, *J* = 8.0 Hz, 2 Hz , 4H), 7.71 (d, *J* = 7.0 Hz, 2H), 7.86 (s, 2H), 8.06 (dt, *J* = 8.0 Hz, 2 Hz, 4H). ^{13}C NMR ($CDCl_3$, 125 MHz) δ(ppm) 117.1, 123.6, 127.1, 128.6, 129.2, 131.9, 138.2, 156.4.

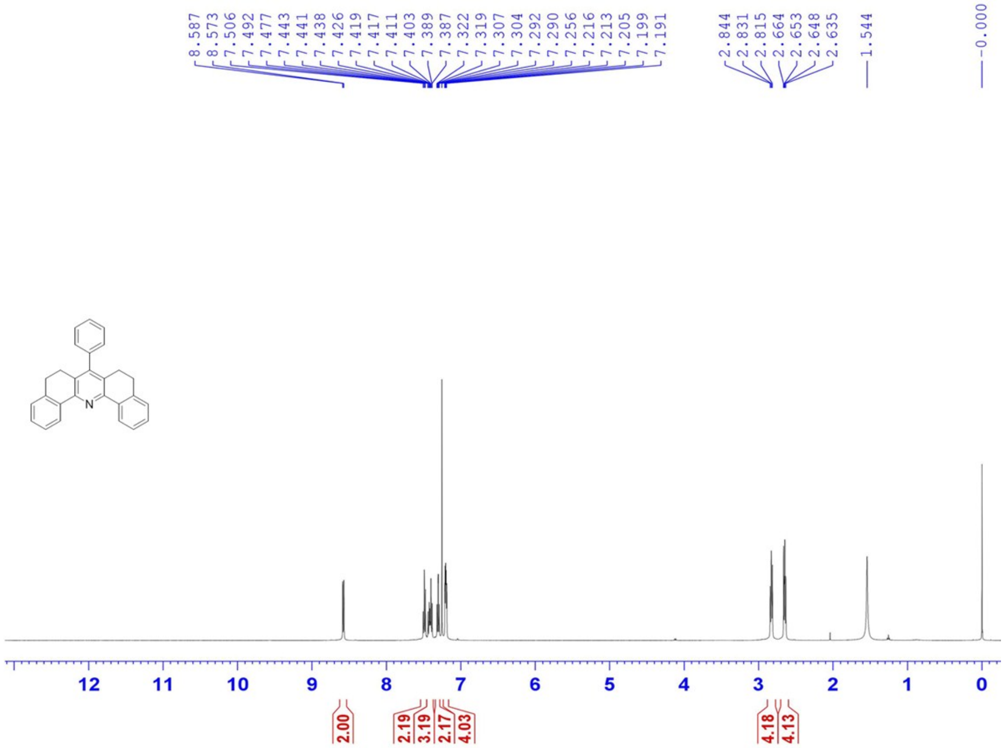

Fig.S20. ^{1}H-NMR spectra of 7-phenyl-5,6,8,9-tetrahydrodibenzo[*c,h*]acridine.

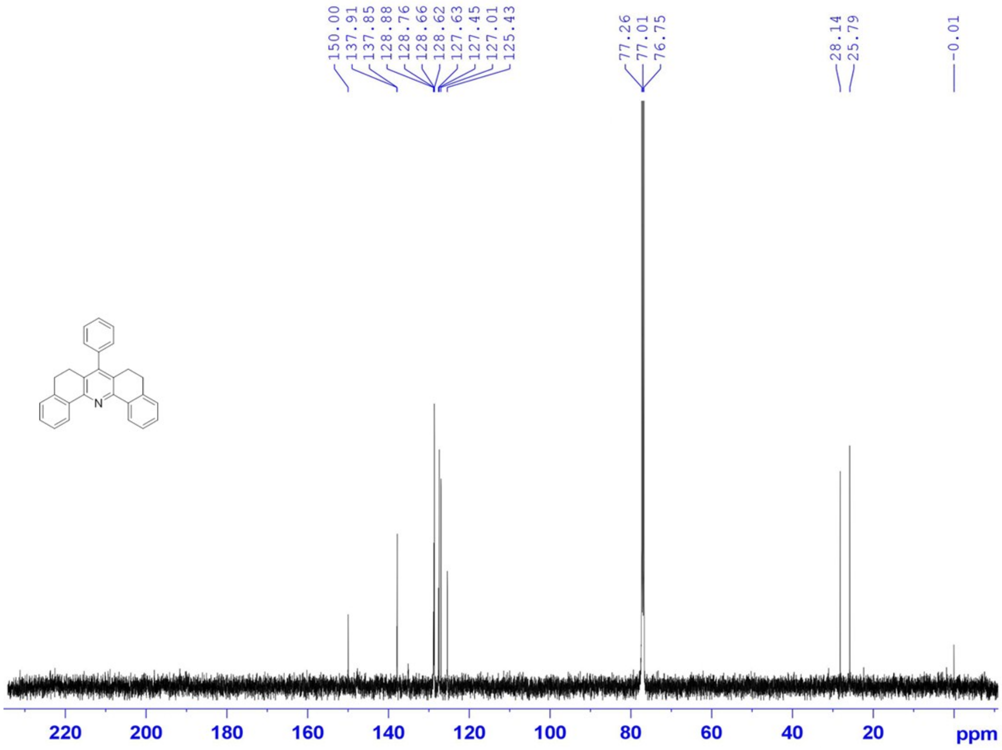

Fig.S21. ^{13}C-NMR spectra of 7-phenyl-5,6,8,9-tetrahydrodibenzo[*c,h*]acridine.

Characterization data for 7-phenyl-5,6,8,9-tetrahydrodibenzo[*c,h*]acridine

Prepared as shown in the general experimental procedure and purified on silica gel (230-400 mesh or 37-63 μm, ethyl acetate/hexane = 1:30 (v./v.), TLC silica gel 60 F_{254}, R_f = 0.5): Yellow solid, 69 % yield (49.5 mg). ^{1}H-NMR (500 MHz, $CDCl_3$) δ(ppm) 2.63 – 2.66 (m, 4H), 2.81 – 2.84 (m, 4H), 7.19 – 7.21 (m, 4H), 7.29 (td, *J* = 7.5 Hz, 1.5 Hz, 2H), 7.38 – 7.44 (m, 3H), 7.47 – 7.50 (m, 2H), 8.57 (d, *J* = 7.0 Hz, 2H). ^{13}C NMR (CDCl3, 125 MHz) δ(ppm) 25.7, 28.1, 125.4, 127.0, 127.4, 127.6 128.6, 128.6 128.7, 128.8, 137.8, 137.9, 150.0.

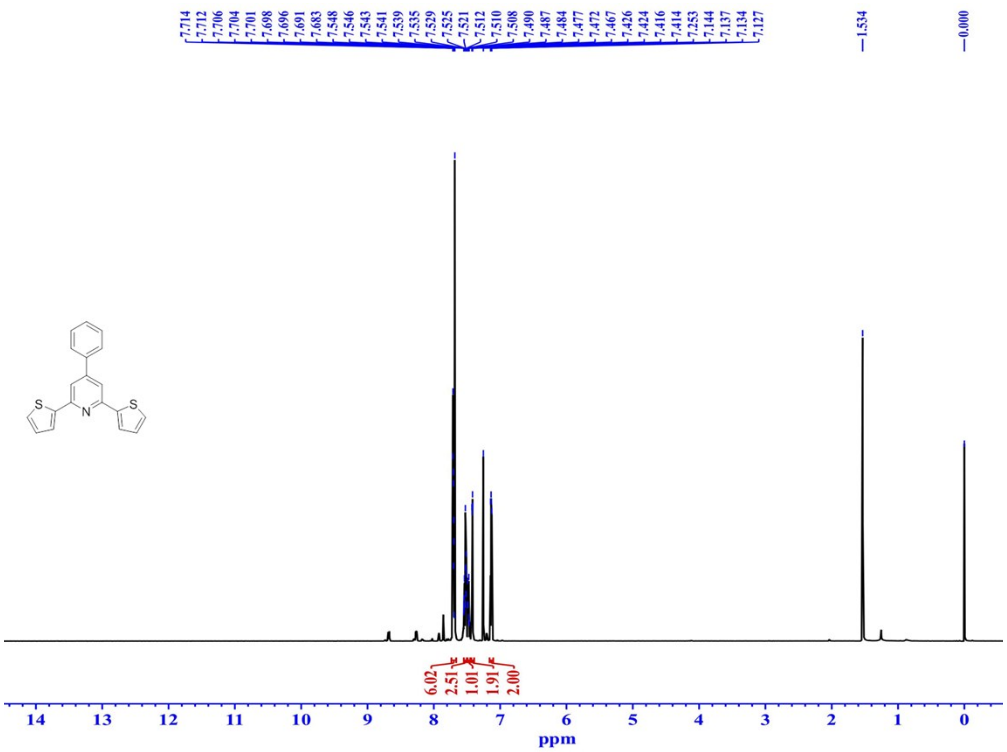

Fig.S22. ^{1}H-NMR spectra of 4-phenyl-2,6-di(thiophen-2-yl)pyridine.

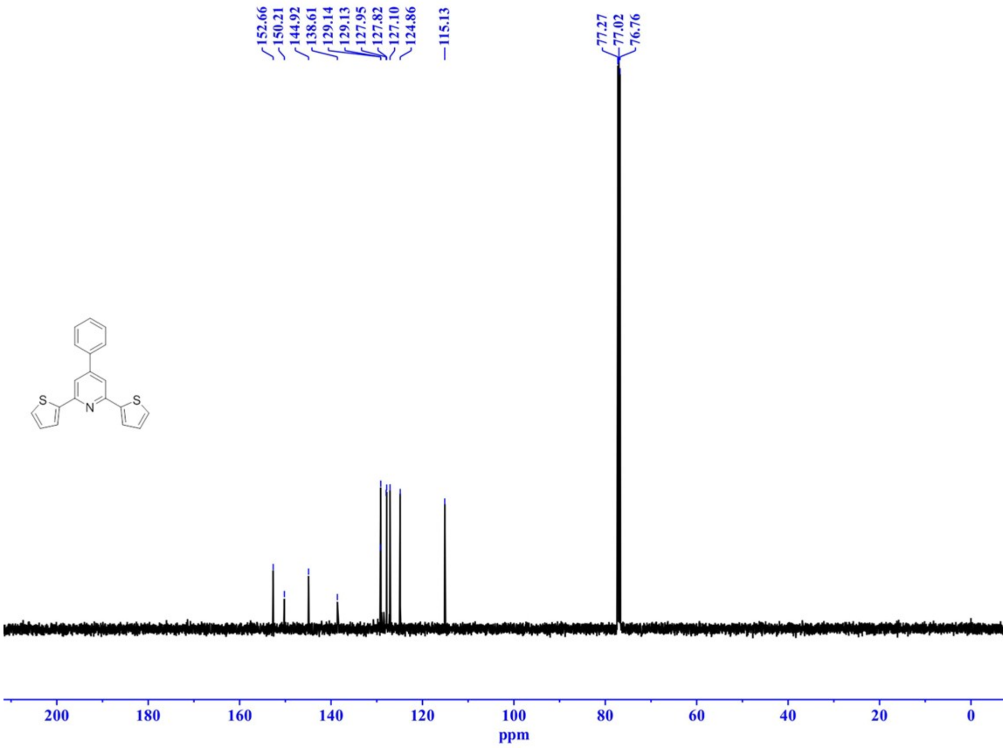

Fig.S23. ^{13}C-NMR spectra of 4-phenyl-2,6-di(thiophen-2-yl)pyridine.

Characterization data for 4-phenyl-2,6-di(thiophen-2-yl)pyridine

Prepared as shown in the general experimental procedure and purified on silica gel (230-400 mesh or 37-63 μm, ethyl acetate/hexane = 1:20 (v./v.), TLC silica gel 60 F_{254}, R_f = 0.4): Yellow solid, 72% yield (45.9 mg). ^{1}H-NMR (500 MHz, $CDCl_3$) δ(ppm) 7.12 – 7.14 (m, 2H), 7.41 (d, *J* = 5.0 Hz, 1Hz, 2H), 7.45 (tt, *J* = 7.0 Hz, 1H), 7.50 – 7.54 (m, 2H), 7.68 – 7.71 (m, 6H). ^{13}C NMR (CDCl3, 125 MHz) δ(ppm) 115.1, 124.8, 127.1, 127.8, 127.9, 129.1, 129.1, 138.6, 144.9, 150.2, 152.6.

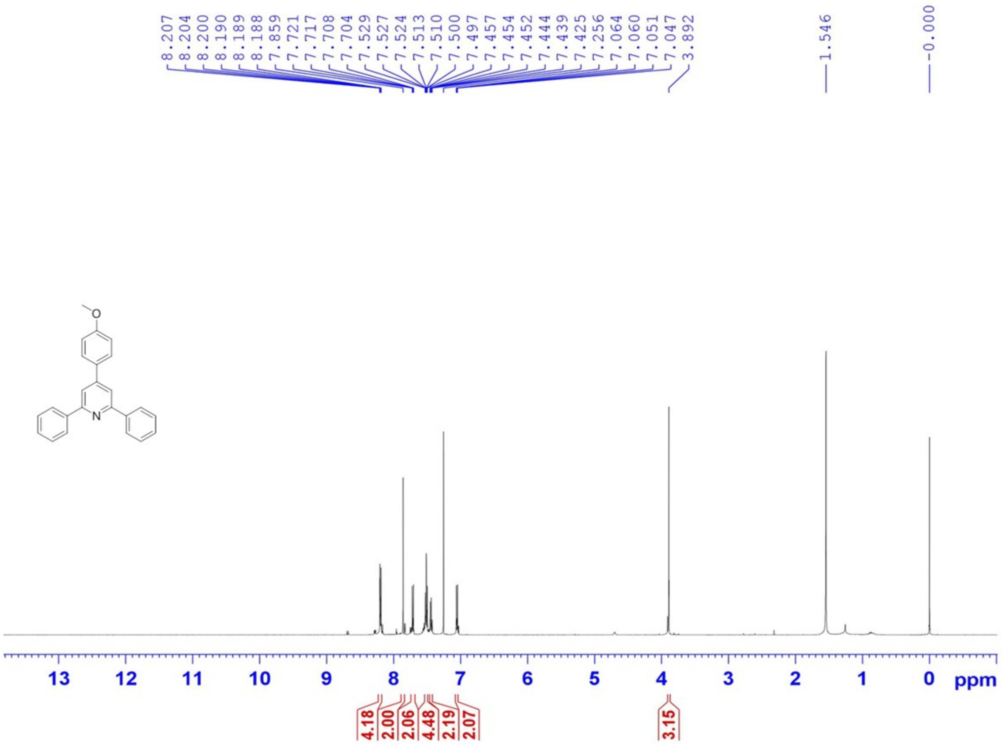

Fig.S24. ¹H-NMR spectra of 4-(4-methoxyphenyl)-2,6-diphenylpyridine.

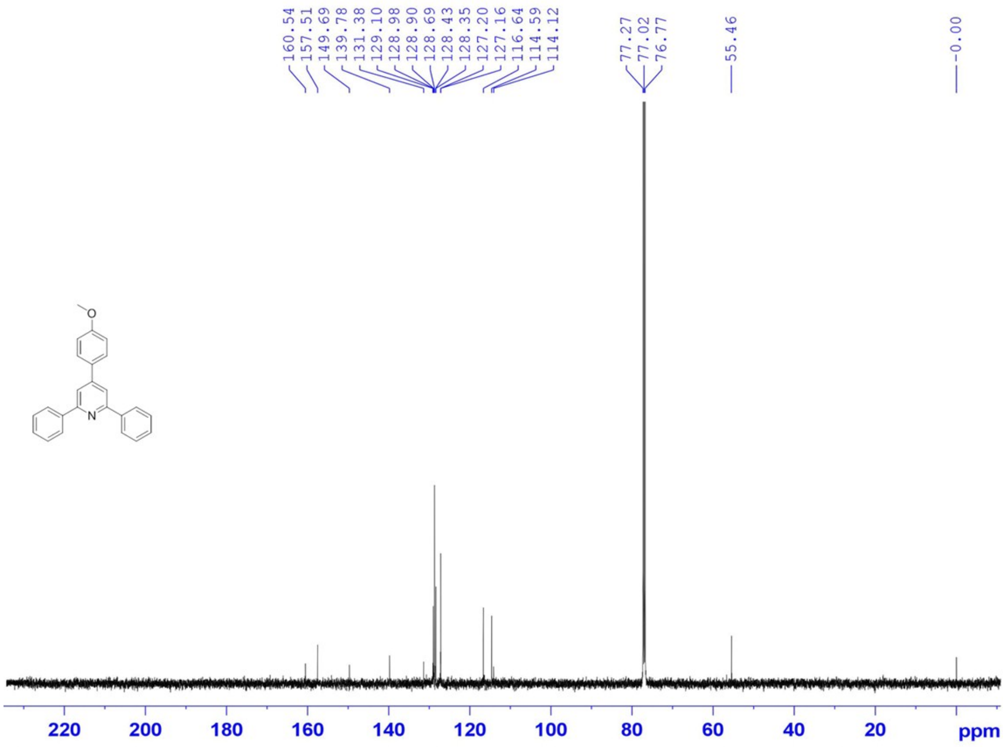

Fig.S25. ^{13}C-NMR spectra of 4-(4-methoxyphenyl)-2,6-diphenylpyridine.

Characterization data for 4-(4-methoxyphenyl)-2,6-diphenylpyridine

Prepared as shown in the general experimental procedure and purified on silica gel (230-400 mesh or 37-63 μm, ethyl acetate/hexane = 1:20 (v./v.), TLC silica gel 60 F_{254}, R_f = 0.4): Yellow solid, 54% yield (36.4 mg). ^{1}H-NMR (500 MHz, $CDCl_3$) δ(ppm) 3.89 (s, 3H), 7.04 (d, *J* = 8.5 Hz, 2H), 7.42 (t, *J* = 7.5 Hz, 2H), 7.49 (t, *J* = 8.0 Hz, 4H), 7.70 (d, *J* = 8.5 Hz, 2H), 7.85 (s, 2H), 8.18 (d, *J* = 7.5 Hz, 4H). ^{13}C NMR (CDCl3, 125 MHz) δ(ppm) 55.4, 114.1, 114.5, 116.6, 127.1, 127.2, 128.3, 128.4, 128.6, 128.9, 128.9, 129.1, 131.3, 139.7, 149.6, 157.5, 160.5.

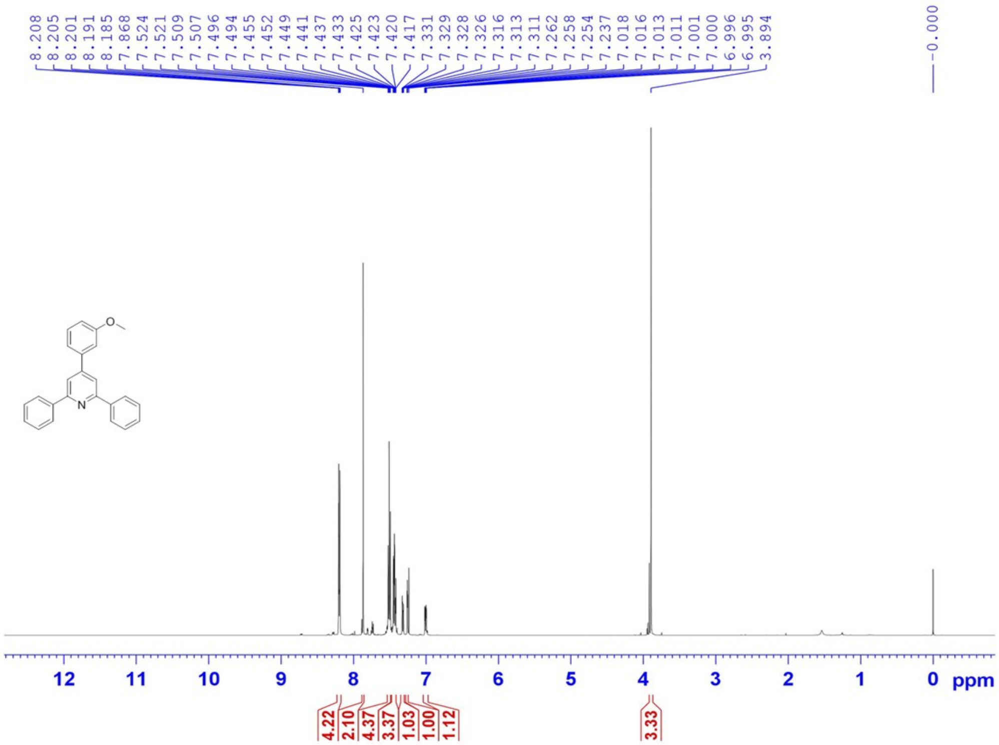

Fig.S26. ^{1}H-NMR spectra of 4-(3-methoxyphenyl)-2,6-diphenylpyridine.

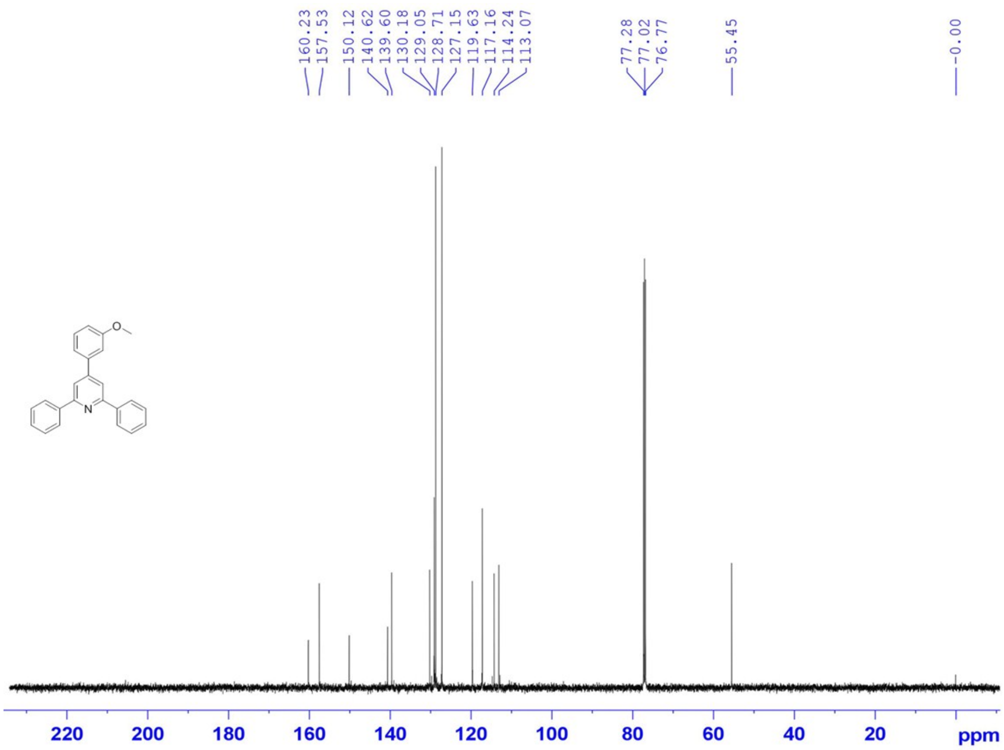

Fig.S27. ^{13}C-NMR spectra of 4-(3-methoxyphenyl)-2,6-diphenylpyridine.

Characterization data for 4-(3-methoxyphenyl)-2,6-diphenylpyridine

Prepared as shown in the general experimental procedure and purified on silica gel (230-400 mesh or 37-63 μm, ethyl acetate/hexane = 1:20 (v./v.), TLC silica gel 60 F_{254}, R_f = 0.4): Yellow solid, 53% yield (35.7 mg). ^1H-NMR (500 MHz, $CDCl_3$) δ(ppm) 3.89 (s, 3H), 6.99 (dd, *J* = 8.0 Hz, 2.0 Hz, 1H), 7.25 (t, *J* = 2.0 Hz, 1H), 7.32 (dt, *J* = 7.5 Hz, 1.5 Hz, 1H), 7.41-7.45 (m, 3H), 7.49 (t, *J* = 7.5 Hz, 4H), 7.86 (s, 2H), 8.18 (d, *J* = 7.0 Hz, 4H).^{13}C NMR (CDCl3, 125 MHz) δ(ppm) 55.4, 113.0, 114.2, 117.1, 119.6, 127.1, 128.7, 129.0, 130.1, 139.6, 140.6, 150.1, 157.5, 160.2.

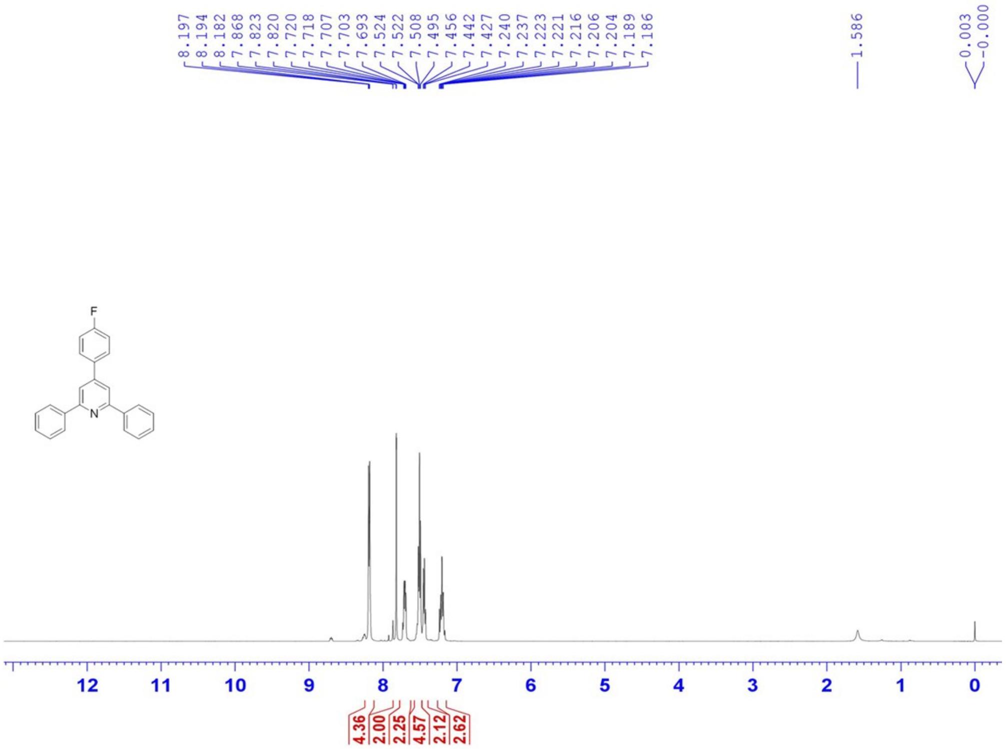

Fig.S28. ^{1}H-NMR spectra of 4-(4-fluorophenyl)-2,6-diphenylpyridine.

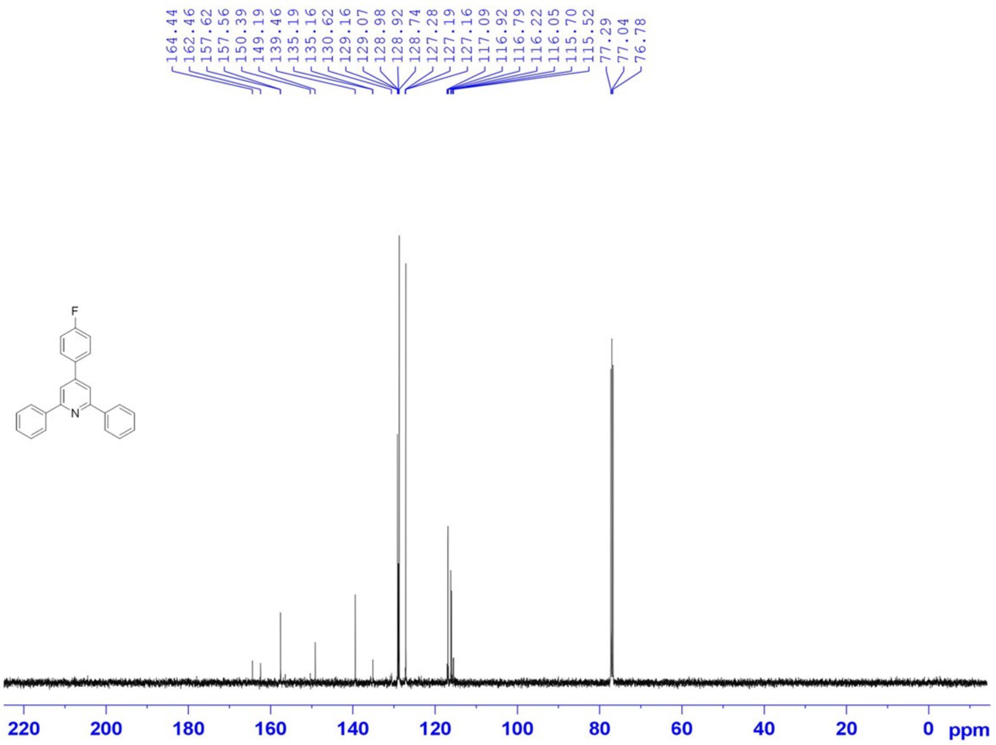

Fig.S29. ^{13}C-NMR spectra of 4-(4-fluorophenyl)-2,6-diphenylpyridine.

Characterization data for 4-(4-fluorophenyl)-2,6-diphenylpyridine

Prepared as shown in the general experimental procedure and purified on silica gel (230-400 mesh or 37-63 μm, ethyl acetate/hexane = 1:20 (v./v.), TLC silica gel 60 F_{254}, R_f = 0.4): Yellow solid, 72 % yield (46.8 mg). ^{1}H-NMR (500 MHz, $CDCl_3$) δ(ppm) 7.18 – 7.24 (m, 2H), 7.42 – 7.45 (m, 2H), 7.49 – 7.52 (m, 4H), 7.69 – 7.72 (m, 2H), 7.82 (s, 2H), 8.18 (d, *J* = 7.5Hz, 4H). ^{13}C NMR ($CDCl_3$, 125 MHz) δ(ppm) 116.0 (d, J_{CF} = 21.2 Hz), 116.7, 127.1, 128.7, 128.9 (d, J_{CF} = 7.5 Hz), 129.1, 135.1, 139.4, 149.1, 157.5, 162.4, 164.4.

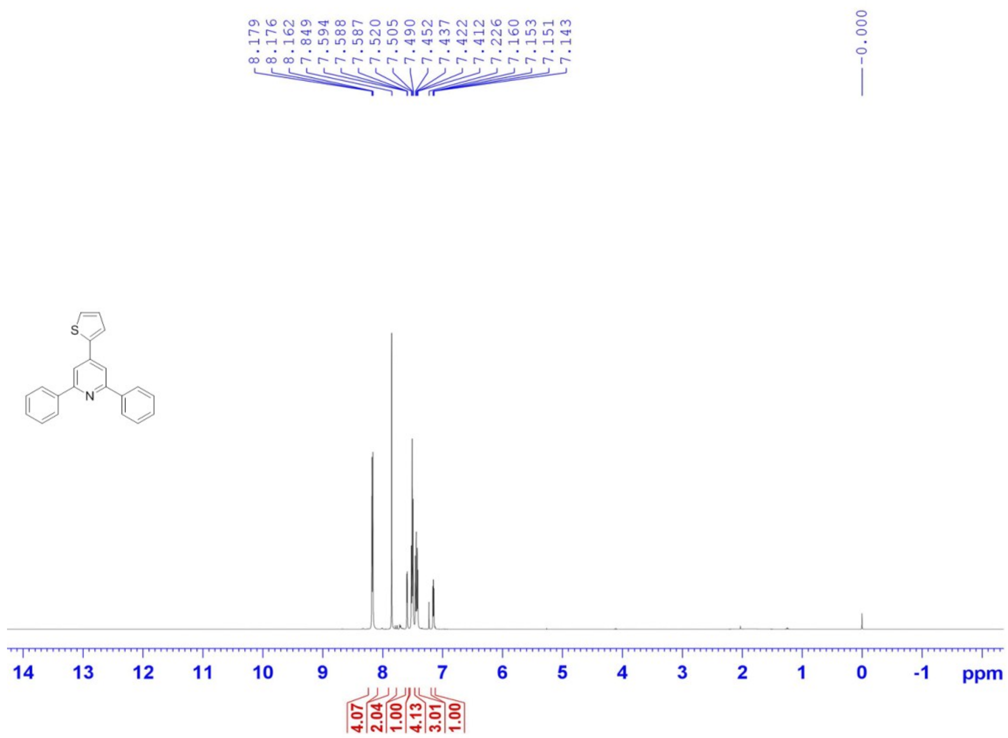

Fig.S30. ^{1}H-NMR spectra of 2,6-diphenyl-4-(thiophen-2-yl)pyridine.

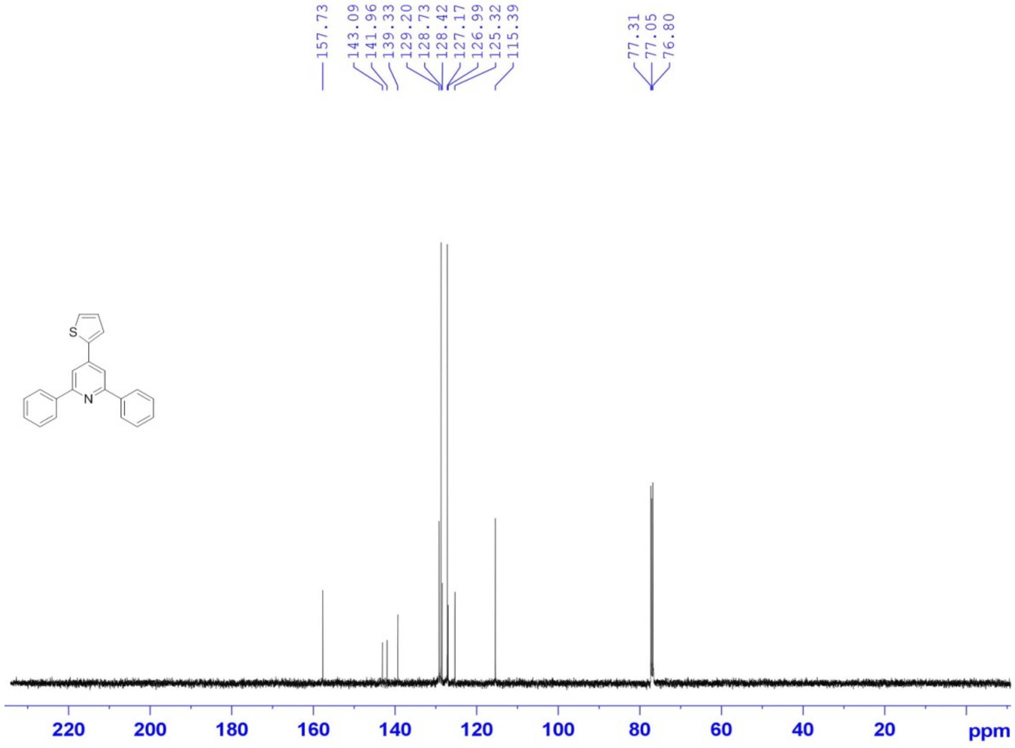

Fig.S31. ^{13}C-NMR spectra of 2,6-diphenyl-4-(thiophen-2-yl)pyridine.

Characterization data for 2,6-diphenyl-4-(thiophen-2-yl)pyridine

Prepared as shown in the general experimental procedure and purified on silica gel (230-400 mesh or 37-63 μm, ethyl acetate/hexane = 1:50 (v./v.), TLC silica gel 60 F_{254}, R_f = 035): White solid, 53% yield (33.2 mg). ^{1}H NMR (500 MHz, $CDCl_3$) δ(ppm) 7.14 – 7.16 (m, 1H), 7.41 – 7.45 (m, 3H), 7.49 – 7.52 (m, 4H), 7.58 (d, *J* = 3.5 Hz, 1H), 7.84 (s, 2H), 8.16 (d, *J* = 7.5 Hz, 4H). ^{13}C NMR ($CDCl_3$, 125 MHz) δ(ppm) 115.3, 125.3, 126.9, 127.1, 128.4, 128.7, 129.2, 139.3, 141.9, 143.0, 157.7.

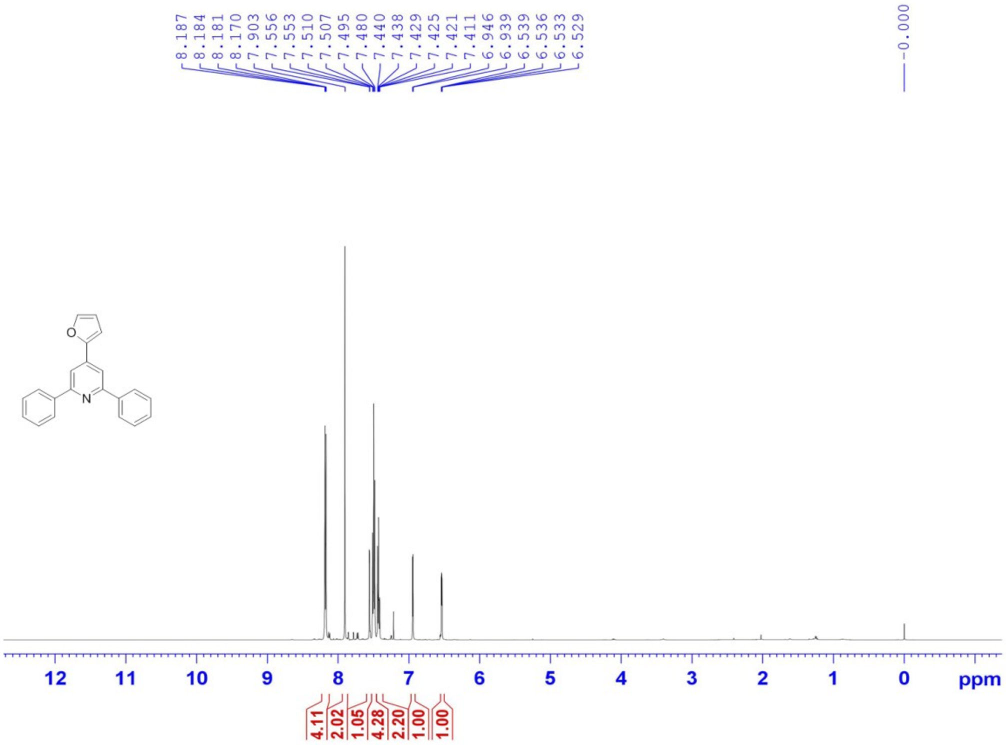

Fig.S32. ^{1}H-NMR spectra of 4-(furan-2-yl)-2,6-diphenylpyridine.

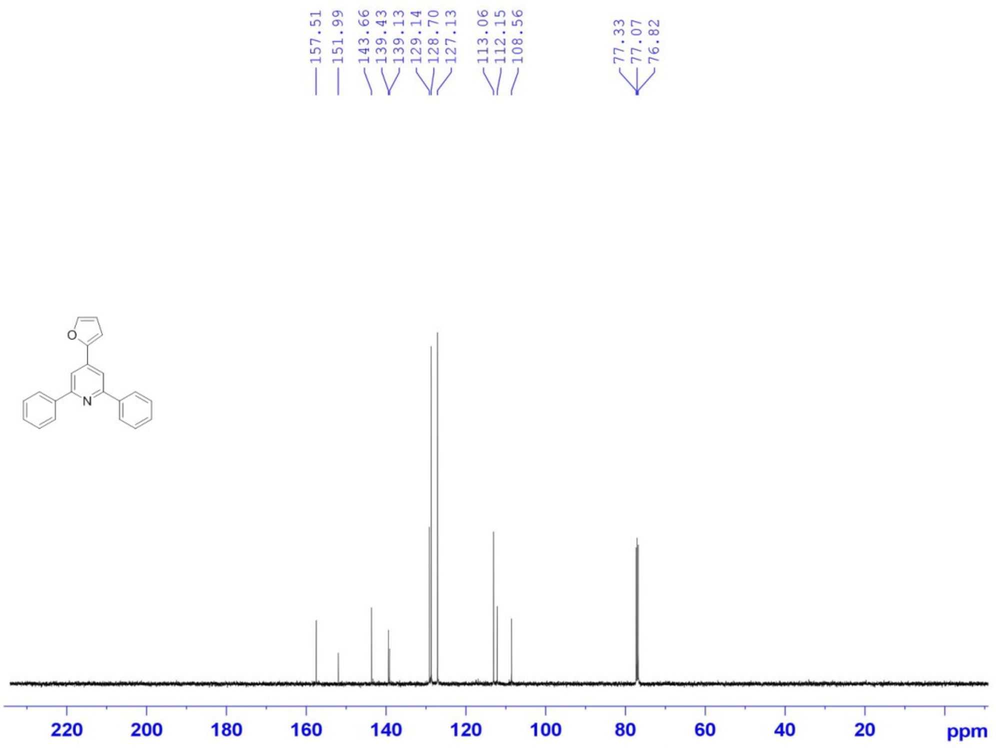

Fig.S33. ^{13}C-NMR spectra of 4-(furan-2-yl)-2,6-diphenylpyridine.

Characterization data for 4-(furan-2-yl)-2,6-diphenylpyridine

Prepared as shown in the general experimental procedure and purified on silica gel (230-400 mesh or 37-63 μm, ethyl acetate/hexane = 1:50 (v./v.), TLC silica gel 60 F_{254}, R_f = 0.35): yellow solid, 59% yield (35.1 mg). ^{1}H NMR (500 MHz, $CDCl_3$) δ(ppm) 6.52 – 6.53 (m, 1H), 6.93 (d, *J* = 3.5 Hz, 1H), 7.41 – 7.44 (m, 2H), 7.48 – 7.51 (m, 4H), 7.53 (d, *J* = 1.5 Hz, 1H), 7.90 (s, 2H), 8.17 (d, *J* = 7.5 Hz, 4H). ^{13}C NMR ($CDCl_3$, 125 MHz) δ(ppm) 108.5, 112.1, 113.0, 127.1, 128.7, 129.1, 139.1, 139.4, 143.6, 151.9, 157.5.

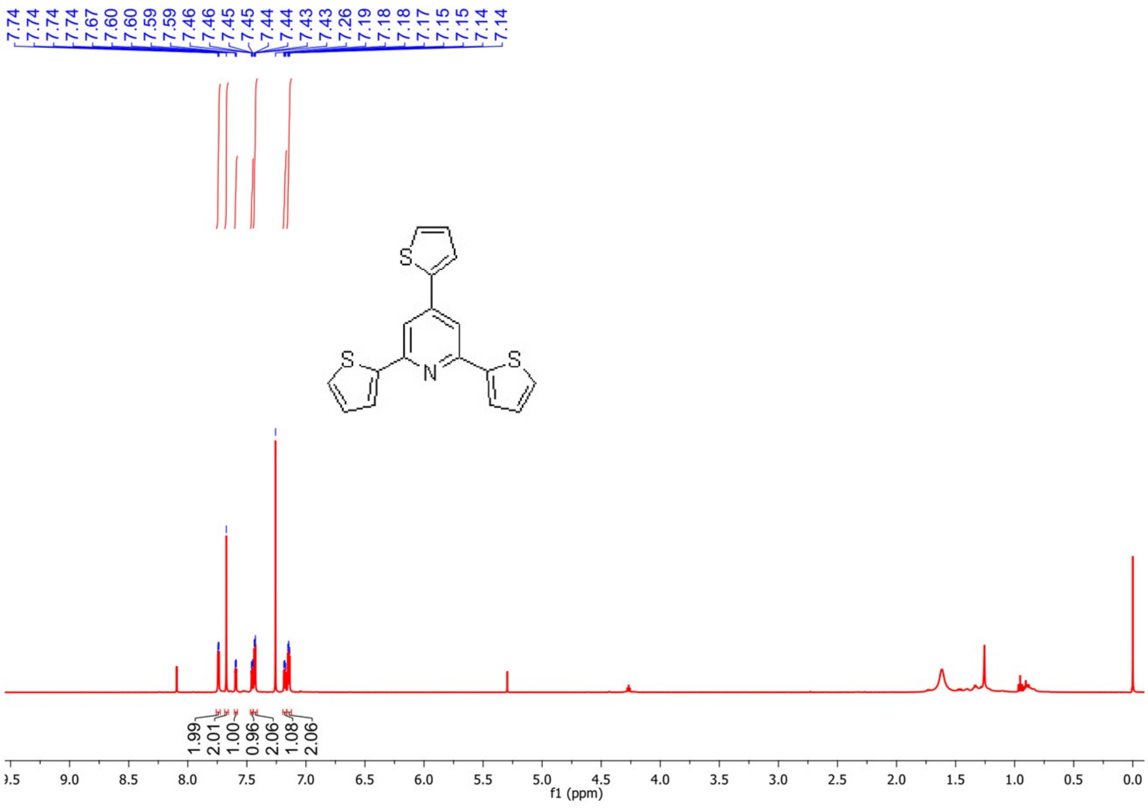

Fig.S34. ^{1}H-NMR spectra of 2,4,6-tri(thiophen-2-yl)pyridine.

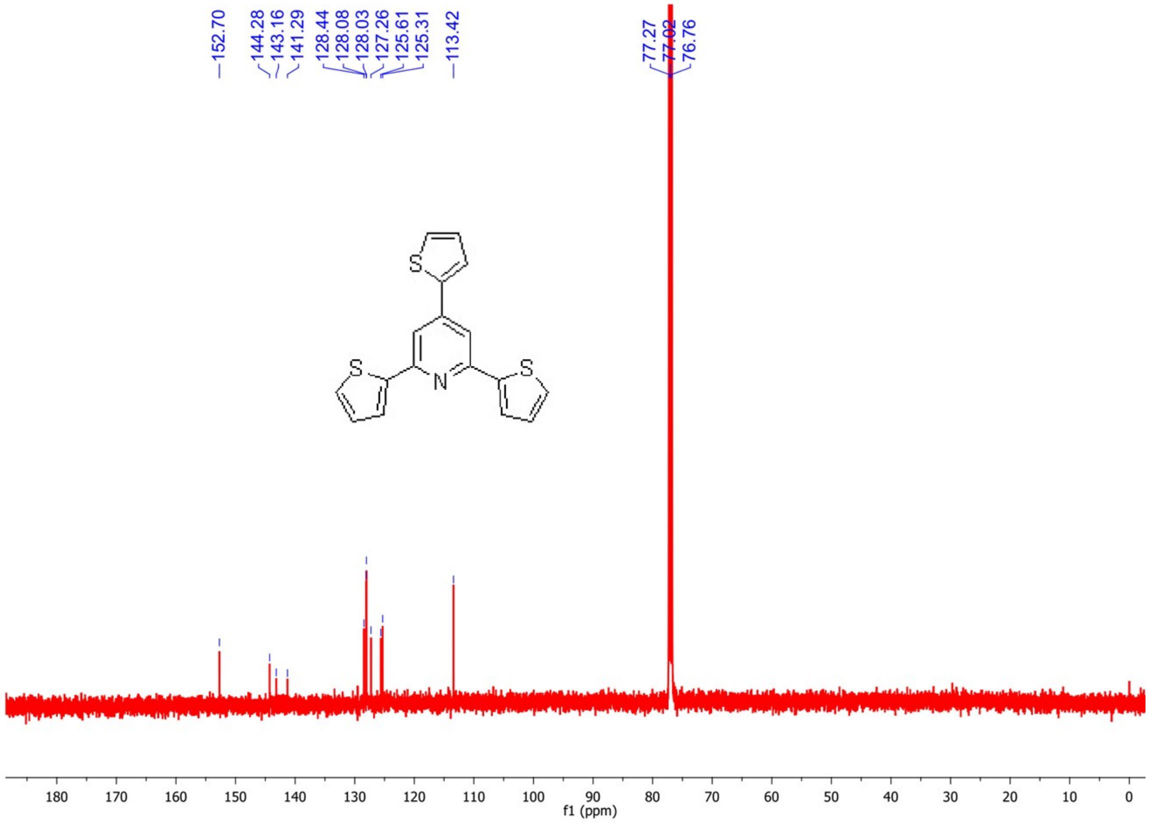

Fig.S35. ^{13}C-NMR spectra of 2,4,6-tri(thiophen-2-yl)pyridine.

Characterization data for 2,4,6-tri(thiophen-2-yl)pyridine

Prepared as shown in the general experimental procedure and purified on silica gel (230-400 mesh or 37-63 μm, ethyl acetate/hexane = 1:50 (v./v.), TLC silica gel 60 F_{254}, R_f = 0.35): yellow solid, 50% yield (32 mg). ^{1}H NMR (500 MHz, $CDCl_3$) δ(ppm) 7.74 (dd, *J* = 3.6, 0.8 Hz, 2H), 7.67 (s, 2H), 7.59 (dd, *J* = 3.6, 1.0 Hz, 1H), 7.46 (dd, *J* = 5.1, 1.0 Hz, 1H), 7.43 (dd, *J* = 5.0, 0.9 Hz, 2H), 7.18 (dd, *J* = 5.0, 3.7 Hz, 1H), 7.14 (dd, *J* = 5.0, 3.7 Hz, 2H). ^{13}C NMR ($CDCl_3$, 125 MHz) δ(ppm) 152.7, 144.3, 143.2, 141.3, 128.4, 128.1, 128.0, 127.3, 125.6, 125.3, 113.4.
